# Electro-Physical Technique for Post-Fabrication Measurements of CMOS Process Layer Thicknesses

**DOI:** 10.6028/jres.112.018

**Published:** 2007-10-01

**Authors:** Janet C. Marshall, P. Thomas Vernier

**Affiliations:** National Institute of Standards and Technology, Gaithersburg, MD 20899-8120; University of Southern California, Marina del Rey, CA 90292

**Keywords:** CMOS, MEMS, nomenclature, platform height, step height, test structures, thickness, Young’s modulus

## Abstract

This paper^1^ presents a combined physical and electrical post-fabrication method for determining the thicknesses of the various layers in a commercial 1.5 μm complementary-metal-oxide-semiconductor (CMOS) foundry process available through MOSIS. Forty-two thickness values are obtained from physical step-height measurements performed on thickness test structures and from electrical measurements of capacitances, sheet resistances, and resistivities. Appropriate expressions, numeric values, and uncertainties for each layer of thickness are presented, along with a systematic nomenclature for interconnect and dielectric thicknesses. However, apparent inconsistencies between several of the physical and electrical results for film thickness suggest that further uncertainty analysis is required and the effects of several assumptions need to be quantified.

## 1. Introduction

POST-fabrication determination of thickness values for the various layers in a complementary-metal-oxide-semiconductor (CMOS) process poses measurement and analytical challenges. Process and lot-specific variations in these quantities are typically tracked indirectly, with no direct physical validation, even though accurate knowledge of interconnect and dielectric layer thicknesses can be critical for modeling and monitoring the behavior of microelectromechanical, radio frequency (RF), and optoelectronic devices, to name a few.

Gate oxide thickness scales with the polysilicon width (or the channel length), junction depth, and supply voltage. Other layer thicknesses not directly related to this active device scaling are increasingly important for circuit behavior. Post-fabrication measurements will facilitate not only process control but also the accuracy of simulations critically dependent on interconnect parasitics in addition to device performance.

Gate oxide thickness is a fundamental CMOS process parameter. A thinner gate oxide enables smaller and faster transistors and critically affects their properties, and a variety of techniques have been developed to provide accurate gate oxide thickness values. Measurement methods include x-ray photoelectron spectroscopy, Auger electron spectroscopy, secondary ion mass spectrometry, Rutherford backscattering, transmission electron microscopy, and spectroscopic ellipsometry [1]. Pull-in [2] and optomechanical [3] techniques are used to measure thicknesses in micro-electromechanical system (MEMS) processes.

Of the above mentioned methods, spectroscopic ellipsometry (SE) [4,5] is the only one that can be used to measure multiple thicknesses on fully fabricated CMOS test chips (a test area of 50 μm by 100 μm is currently required). However, this method does not work for metallization thicker than about 0.04 μm and the layers underlying the thicker metal. In addition, the rough surface of the top glass or nitride layers covering the chips also prevents accurate measurements of underlying layers. But the potential of this technique should be kept in mind as equipment and processes become aligned, especially since the combined standard uncertainty values of the thicknesses found using SE (typically between 0.01 nm and 0.05 nm) are considerably less than those found with the electro-physical technique (0.20 nm to 150 nm for the presented data set). However, the technique presented here provides the thicknesses of all the layers in sufficient accuracy to perform Young’s modulus optimizations.

This work was prompted by the goal of finding the Young’s modulus values for the various layers in a CMOS process [6]. Knowledge of the Young’s modulus values and the residual strain [7] of each layer can lead to calculations of residual stress, which can in turn contribute to circuit design strategies and fabrication and post-processing methods that help increase fabrication yield by reducing the frequency of failures from electromigration, stress migration, and delamination. To find the Young’s modulus values, a test chip was designed and fabricated with 16 distinct CMOS MEMS cantilevers in conjunction with the thickness test structures described in this paper. The resonant frequencies of the cantilevers and the thicknesses of each layer are input into an optimization program to find the Young’s modulus values of the various layers. For the processing run being considered, this paper presents a set of thickness values for use with the optimization.

**Fig. 1:**
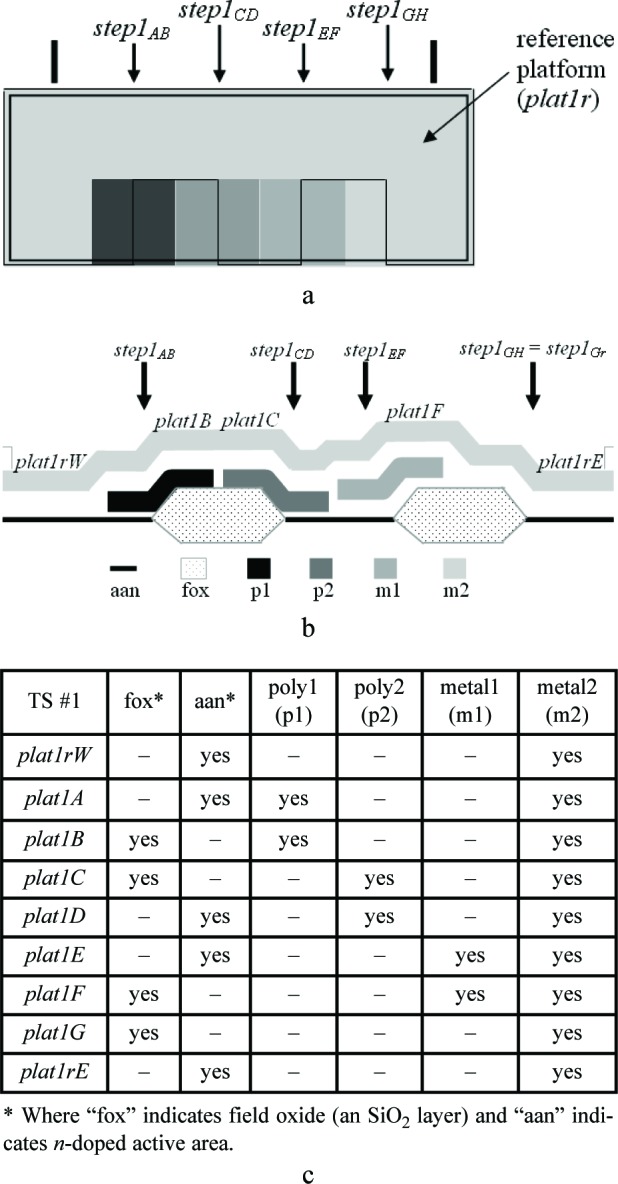
For thickness test structure #1: a) a design rendition (a plan view), b) a cross section (where the uncolored regions beneath the metal2 layer correspond to one or more oxide), and c) prominent features (i.e., field oxide or active area and the interconnect layers) beneath each platform.

In this paper, an electro-physical technique is presented which melds two post-fabrication approaches— physical and electrical—to obtain thickness values. The physical approach uses thickness test structures, such as those shown in Fig. 1. Step-height measurements from these thickness test structures are measured on fully fabricated chips using instruments such as an optical interferometer, a stylus profilometer, an atomic force microscope, and a scanning electron microscope.

The electrical approach utilizes capacitance and sheet resistance measurements that are posted publicly [8] for each MOSIS^2^ multi-project wafer lot. Additional thickness values are extracted from interconnect resistivity values and properties of the silicon dioxide crystal lattice, and by assuming ideal thermal oxidation of silicon [9].

The 1.5 μm (feature size) commercial CMOS process depicted in Fig. 1 can provide 48 distinct combinations of interconnect and dielectric layers over field oxide on *p*-type substrate (*p*-well) and over *n*-type active area (without including contacts and vias). These 48 combinations utilize 10 different layers (4 interconnects, 4 oxides, the glass passivation layer, and a nitride cap that is present on top of the glass layer when the chips are received from MOSIS). These 10 different layers can have 25 different thicknesses depending upon the grown and deposited oxide properties and the etch sequence and effects; however, a set of 42 thickness values will be presented for this process, the extra thicknesses being useful from an analytical standpoint, which will become clearer later. These thicknesses were obtained by the physical approach, the electrical approach, or both.

The calculations presented in this paper and the calculations for the layer thicknesses in other similar processing runs can be performed on-line [10].

## 2. Test Structures, Sample Preparation, and Nomenclature

This section presents the test structures used in this work in Sec. 2.1, the sample preparation in Sec. 2.2, and the nomenclature in Sec. 2.3.

### 2.1 Test Structures

**Fig. 2:**
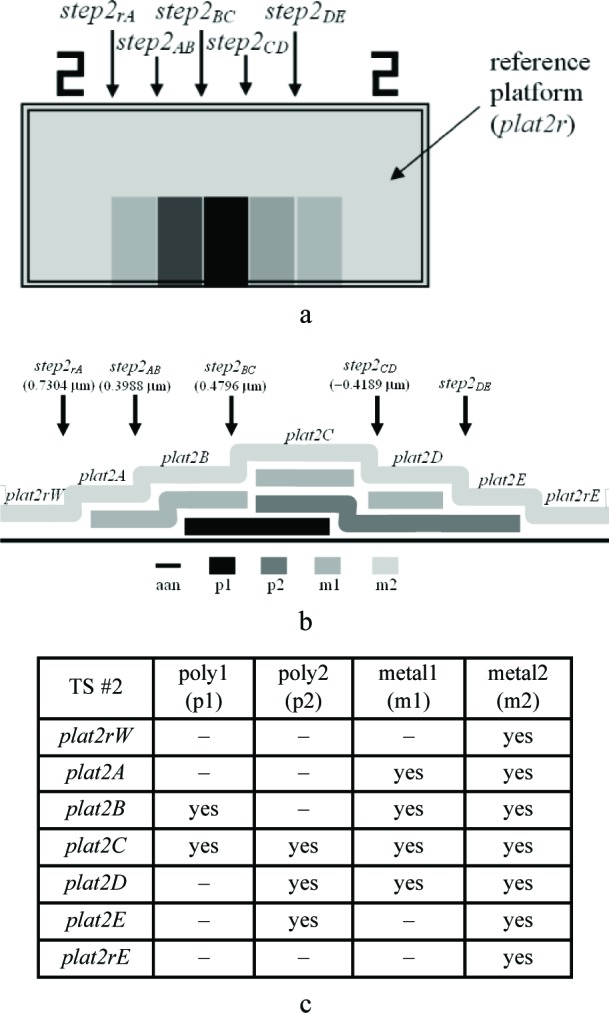
For TS #2: a) a design rendition, b) a cross section (where the uncolored regions beneath the metal2 layer correspond to one or more oxide), and c) the interconnects beneath each platform.

**Fig. 3:**
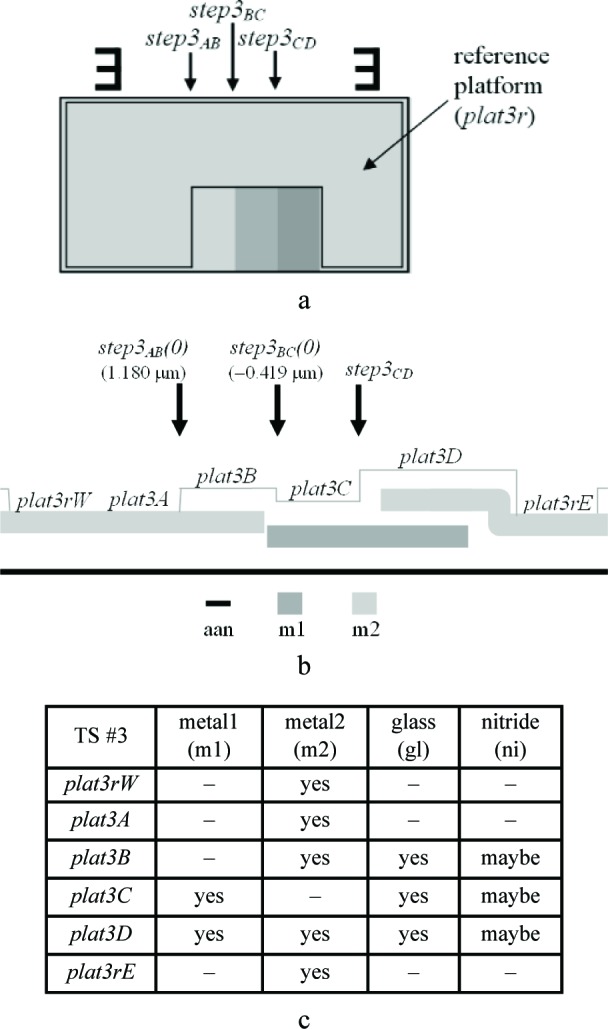
For TS #3: a) a design rendition, b) a cross section (where the uncolored regions above the active area correspond to one or more oxide including the glass layer and nitride cap), and c) prominent features beneath each platform.

Three thickness test structures are used in this work. Test structure #1 (TS #1) is presented in Fig. 1, test structure #2 (TS #2) is presented in Fig. 2, and test structure #3 (TS #3) is presented in Fig. 3. These figures include the design renditions, the corresponding cross sections, and prominent features beneath each platform.

### 2.2 Sample Preparation

The design files are submitted to MOSIS [8] for fabrication on a commercial CMOS process. After fabrication, MOSIS sends approximately 15 chips to each participant in that particular multi-project processing run. In this paper, one of these chips is used to obtain step-height measurements from two of the thickness test structures (i.e., TS #1 and TS #2) presented in Sec. 2.1, and two additional chips are used to obtain step-height measurements from TS #3. Therefore, three of the fifteen test chips received from MOSIS are used for step-height measurements.

A non-contact optical interferometer is used to take the step-height measurements in this paper. Since the top metal2 layer (with a thickness of approximately 1.0 μm) in TS #1 and in TS #2 is reflective (a requirement for optical interferometry), measurements are taken on these test structures on one of the three chips received directly from MOSIS, before any post processing is done.

In TS #3, the top glass or nitride cap layer is not reflective so for interferometric measurements, additional post-processing is required. On the second of the three chips is evaporated approximately 8 nm of chromium followed by approximately 150 nm of gold before measurements are taken on TS #3. The chromium helps the gold adhere to the chip while the gold coverage provides a smooth, top reflective surface to ensure an accurate interferometric measurement. These additional layers are assumed to have a uniform thickness across the test chip and, as such, do not enter into the calculations. Therefore, these layers will not appear in the pertinent figures throughout this paper.

The third of the three chips undergoes a XeF_2_ etch [11] until the nitride cap layer is lifted off the underlying glass layer. Then, a thin chromium layer and a thin gold layer are evaporated on the chip as specified in the previous paragraph before additional measurements are taken on TS #3.

### 2.3 Nomenclature

**Fig. 4:**
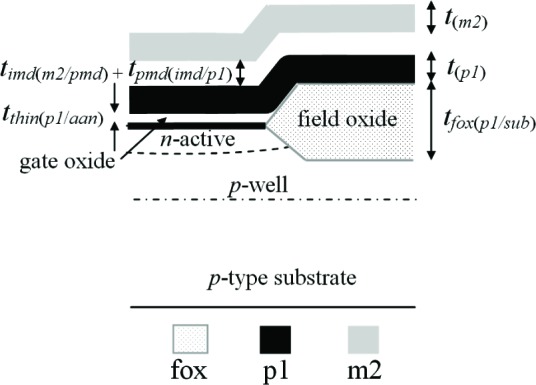
A more complete cross section.

Two types of symbols (#1 to #7 and #8 to #25 of Table 1) have been developed to keep track of the 42 different thickness values; 25 of which are given in Table 1.^3^ The first type of symbol (for interconnects, glass, and the nitride cap) can be represented by the thickness designations given in #1 to #7. The second type of symbol (for the oxide dielectric layers) can be represented by the thickness designations given in #8 to #25. The thickness designations for both these symbols start with the letter “*t*” and are followed by one or more subscripts. Refer to Fig. 4 for familiarization with a more detailed cross section and the use of some of these symbols.

The first type of symbol (in #1 to #7 of Table 1) is for the interconnects, glass, and nitride cap. As an example, consider the poly2 thickness given by the thickness designation *t_(p2)_* in #3. The shorthand notation for the layer (namely, p2) is given in parentheses in column 3. This parenthesized shorthand notation becomes the subscript of “*t*” in the thickness designation (as shown in column 2).

The second type of symbol (in #8 to #25 of Table 1) is used for all the oxide layers below the glass layer. As an example, the thickness of the oxide layer between p2 and p1 (#13 in Table 1) is given as *t_thin(p2/p1)_*. The first subscript “*thin*” indicates what oxide is being considered. There are four possibilities for these oxides: “*Fox*” stands for field oxide; “*thin*” is for the thin, thermal oxide between a polysilicon layer and the active area, or between the two polysilicon layers; “*pmd*” is for the deposited oxide before the m1 deposition (also called PMD or poly-to-metal dielectric); and “*imd*” is for the deposited oxide after the m1 deposition (also called IMD or inter-metal dielectric).

The second subscript (given in parentheses) specifies the two layers between which the oxide resides with the topmost layer specified first. Therefore, *t_thin(p2/p1)_* indicates there is a thin, thermal oxide between the p2 and p1 layers. Given the designation *t_fox(pmd/sub)_*, this refers to the field oxide thickness between the deposited PMD oxide and the substrate, *t_pmd(m1/fox)_* refers to the deposited PMD oxide thickness between m1 and the field oxide, and *t_thin(p1/aan)_* refers to the thin, thermal oxide thickness between p1 and the *n*-doped active area. (In this paper, we will only be dealing with *n*-doped active area.)

The third subscript is optional. If a subscripted “*phys*” follows the thickness symbol [such as in *t_fox(p2/sub)phys_*], it implies that the thickness is found using the physical approach. The subscript “*elec*” would imply the electrical approach is used to determine the thickness.

Additional subscripts will be presented in the following sections.

## 3. The Physical Approach

**Fig. 5:**
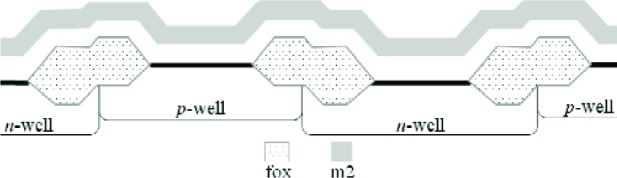
A possible topographical configuration using both wells.

The first approach to be presented is the physical approach, which uses measurements from thickness test structures. The design of a sample thickness test structure (TS) is given in Fig. 1a, with its cross section given in Fig. 1b. The labels on the arrows are the same in Fig. 1a and Fig. 1b, indicating that the locations correspond. These arrows locate the steps where select measurements are taken. As indicated in Fig. 1b, specific design layers comprise the lower and upper platform of each step. The prominent layers included beneath each platform are given in Fig. 1c. Each platform is 50 μm long and 100 μm wide except for the reference platforms on either end of the test structure, which are each 100 μm long. Therefore, each platform consists of a flat spot with sufficient area to obtain a height value. The test structure occupies a *p*-well region of the wafer and does not include *n*-well regions. (MOSIS generates a *p*-well wherever there is no *n*-well specified.) This eliminates possible field oxide thickness variations (as shown in Fig. 5) that can be associated with using different well types.

As can be seen in Fig. 5, the height of the active area in the *p*-well region is different than the height of the active area in the *n*-well region. For the process in this figure, the *p*-well region is protected during the processing of the *n*-well region. This creates a field oxide thickness that is dependent upon which well it is in. The deposited PMD and IMD oxides are typically thicker over lower topographical areas. Assuming the processing in Fig. 5 to be correct, with the active area in the *p*-well region being higher than the active area in the *n*-well region, the deposited PMD and IMD oxide thicknesses would be expected to be thicker over the *n*-well regions than the corresponding thicknesses over the *p*-well regions. For the measurements described in this paper, all of the designs and measurements are over *p*-well regions.

The thickness test structures were fabricated, post-processed as specified in Sec. 2.2 as necessary, and measured. For the test structure in Fig. 1a, four step-height measurements were initially taken, namely, *step1_AB_*, *step1_CD_*, *step1_EF_*, and *step1_GH_* as depicted in Fig. 1b. If two or more different step-height measurements could be taken to determine a particular thickness value, the test structure is chosen that produces the lowest value for the combined standard uncertainty, *u*_c_ (which is comparable to the estimated standard deviation). Note that we are not considering test structures that include contacts or vias, since huge contacts and vias (a requirement for the thickness test structures) are not allowed in chemical mechanical planarization (CMP) processes, which is the typical process for 0.25 μm feature-size processes and below. These processes have density rules where the density and fill must be balanced on all layers, which would not be possible in huge contact and via areas. Therefore, to be compatible with smaller feature-sized processes, this analysis only includes CMP-compatible thickness test structures.

A step consists of two platforms. A measurement of the height of one of the platforms is called a platform-height measurement. The height of these platforms is measured with respect to the reference platform, such as *plat1r* shown in Fig. 1a, for TS #1. Table 2 gives platform-height measurements and their uncertainties as measured with an optical interferometer. In this table, a reference platform is specified with the symbol “*platNrD*,” as shown in #1 and #9, where *N* is the test structure number (“*1*,” “*2*,” “*3*,” etc.), *r* indicates it is from a reference platform, and the optional letter *D* directionally indicates which reference platform (using the compass indicators “*N*,” “*S*,” “*E*,” or “*W*” where “*N*” refers to the reference platform designed closest to the top of the chip). Therefore, as shown in Fig. 1b, *plat1rW* is the leftmost reference platform and *plat1rE* is the rightmost reference platform. The remaining platforms in Table 2^4^ are specified with the symbol “*platNX*” where *N* is the test structure number (“*1*,” “*2*,” “*3*,” etc.) and *X* is the capital letter associated with the platform (“*A*,” “*B*,” “*C*,” etc.) as lettered starting with “*A*” for the platform closest to *platNrW* or *platNrS*. Therefore, *plat1C* shown in Fig. 1b, is the third platform from *plat1rW*. In Table 2, references are made to entries in Table 3 and 4 to indicate the thicknesses of the layers beneath the platforms of each step.

Tables 3 and 4 give the thickness layer combinations for the 48 *p*-well designs over field oxide and *n*-doped active area, respectively, that do not include contacts and vias. In these tables, the possible layers that can be included are listed as column headings. These column headings are listed in the order in which they appear after fabrication with the bottommost layer given first. As such, there are two occurrences of “thin” in Table 4, corresponding to the thin, thermal oxide between a polysilicon layer and the active area, and between the two polysilicon layers. If the layer is included for the particular instance, its thickness designation is specified in the table. Therefore, for instance #9 in Table 3, to obtain the thickness of all the oxides between the substrate and m2, you would add *t_fox(pmd/sub)_*, *t_pmd(imd/fox)_*, and *t_imd(m2/pmd)_*. Many of the thicknesses in Tables 3 and 4 will be used in calculations throughout this paper.

Table 5 gives the step-height measurements and their uncertainties as measured with an optical interferometer. A step-height measurement involves subtracting one platform-height measurement from another, if two different 3-D data sets are used to obtain the platform-height measurements. (Consult the appendix [12] for details.) For the symbol “*step1_CD_*” in Table 5, and shown in Fig. 1b, the number following “*step*” refers to the test structure number it was taken from. The sub-scripted capital letters refer to the two platforms, in this case, *plat1C* and *plat1D*, involved in the measurement. Therefore, *step1_CD_* has *plat1C* on its left and *plat1D* on its right. See the appendix [12] for measurement and calculation specifics; however, if two different 3-D data sets are used to obtain *plat1C* and *plat1D*, the following calculation is used to obtain *step1_CD_*:^5^

$$\begin{array}{l} step1_{CD}=plat1D-plat1C\\ \;=0.4467-0.8763=-0.4296\mu m. \end{array}$$ (1)

Notice that *step1_CD_* is a negative number because it can be viewed as a step “down” when going from left to right. If a lower case “*r*” appears in place of one of the capital letters (as shown in Fig. 1b for *step1_Gr_*), it indicates that the step includes one of the reference platforms. Additional step-height measurements can be found in Table 5. (Note that steps do not need to involve adjacent platforms.) In Secs. 5 and 6, these step-height measurements will be used with layer thicknesses below the pertinent platforms to determine key thicknesses for a possible comparison with those found using the electrical approach.

## 4. The Electrical Approach

The second approach combines the capacitances and sheet resistances that MOSIS reports for each lot (included in the on-line details that MOSIS posts publicly [8]) with process-specific and extracted values for resistivities and oxide thicknesses. These values are used to determine 38 thicknesses, most of which can be directly compared with the physical approach.

This electrical approach is comprised of four components. The first three components will be presented in this section. First, the dielectric^6^ thicknesses are obtained from capacitances, then conductive layer thicknesses are obtained from sheet resistances and resistivities, and finally oxide thicknesses are obtained via the equating of similar oxides. These three components will be presented in the following three sections. The fourth component is the determination of some field oxide thicknesses and *t_(p1_*_′_*_)_*, which utilize crystal lattice calculations.^7^ This component will be integrated with the physical approach in Sec. 5.

### 4.1 Dielectric Thicknesses from Capacitances

The dielectric thicknesses given in Table 6 are obtained from capacitances using the formula

$$t=\varepsilon_{SiO_{2}}/C_{a}$$ (2)

where *t* is the thickness in micrometers, $\varepsilon_{SiO_{2}}$ is the permittivity of SiO_2_ {which equals the dielectric constant of SiO_2_ (3.9) times the vacuum permittivity (8.85 aF/μm) [13]} which equals 34.5 aF/μm, and *C_a_* is the capacitance per unit area in attofarads per square micrometer, for which the fringing capacitance and stray capacitance have been removed [8]. Note in this table that some of the thickness calculations are for combinations of field oxide, PMD, and IMD. The equation for the thickness combined standard uncertainty (*u_c_*) calculations, resulting in the values given in the last column of Tables 6, is presented in the appendix.

### 4.2 Conductive Layer Thicknesses From Sheet Resistances and Resistivities

Values for conductive layer thicknesses are obtained from sheet resistances and resistivities. The interconnect sheet resistance, *R_s_*, values for a 1.5 μm commercial CMOS foundry processing run are given in the third column in Table 7 as obtained from MOSIS. The standard deviations, *σ_Rs_*, of the MOSIS-supplied sheet resistance values are given in the fourth column. The resistivities, *ρ*, in the fifth column are averages derived from measurements at MOSIS on multiple wafer lots. The standard deviations, *σ_ρ_*, of the resistivities in the sixth column, are assumed to be 0.1 Ω μm for p1 and p2 and 0.001 Ω μm for m1 and m2 (which is the uncertainty of the last digit of the resistivities). The thicknesses, in the seventh column, are calculated using the formula

$$t=\rho /R_{s}.$$ (3)

The equation used to calculate the thickness combined standard uncertainty (*u_c_*) values given in the last column is presented in the appendix.

### 4.3 Thicknesses From Equating Similar Oxides

Table 6 includes entries with multiple oxides. These entries correspond to #6, #10, #11, #12, and #13. Additional thicknesses can be obtained by separating these oxides. In order to do that, we equate similar oxides between platforms assuming the layer on top is the same. The layer underneath can be different. This will become clearer as we examine the following four assumptions that apply to interconnects or oxides when going from one platform to the next.

First, it is assumed that the thicknesses of the interconnect layers do not vary with topography, except at steps. Second, it is assumed that the deposited PMD and/or IMD oxide thicknesses sandwiched between similar layers do not change in thickness as a function of topography, except at steps. For example, the m2-tom1 oxide thickness over active area is assumed to be equal to the m2-to-m1 oxide thickness over field oxide. This is contrary to the belief that the deposited oxides are thicker over lower topographical areas. However, contradictory evidence was instrumental in precipitating this assumption. This can be seen by comparing the values for #7 and #8 in Table 6. Number 7, corresponding to *t_pmd(m1/aan)elec_*, is over a lower topographical area than #8, corresponding to *t_pmd(m1/p1)elec_*, and yet the thickness value for #7 is less than the thickness value for #8. In this paper, we do not equate *t_pmd(m1/aan)elec_* with *t_pmd(m1/p1)elec_* but observe that their values appear to be switched if the deposited oxides are indeed thicker over lower topographical areas. Therefore, as applied to the m2-to-m1 oxide thickness, to avoid the possibility of amplifying an error in the ensuing calculations, the m2-to-m1 oxide thickness over active area is assumed to be equal to the m2-to-m1 oxide thickness over field oxide.

**Fig. 6:**
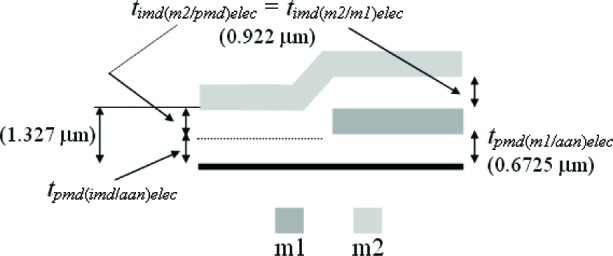
A cross section for equating two IMD thicknesses.

The third assumption involves the IMD oxide shown in Fig. 6. The thickness values given in this figure were taken from Table 6. The PMD and IMD oxide thicknesses on the left hand side of this figure [namely, *t_pmd(imd/aan)elec_* and *t_imd(m2/pmd)elec_*] can be separated if it is assumed that the IMD oxide thicknesses in this figure are equated as follows:

$$t_{imd\left(m2/pmd\right)elec}=t_{imd\left(m2/m1\right)elec}=0.922\mu m.$$ (4)

Therefore, we can calculate the only unknown oxide thickness in Fig. 6 (in order to separate or obtain thickness values for the two oxide thicknesses in #11 of Table 6) as follows:

$$\begin{array}{l} t_{pmd\left(imd/ann\right)elec}={\left[t_{pmd\left(imd/ann\right)}+t_{imd\left(m2/pmd\right)}\right]}_{elec}-t_{imd\left(m2/pmd\right)elec}\\ \;=1.327-0.922=0.405\mu m. \end{array}$$ (5)

In other words, the m2-to-active area thickness given by [*t_pmd(imd/aan)_* + *t_imd(m2/pmd)_*]*_elec_* minus the IMD oxide thickness between m2 and the PMD oxide [*_timd(m2/pmd)elec_*] is equal to the only unknown oxide thickness in Fig. 6, *t**_pmd(imd/aan)elec_*. Referring to this figure, this value of 0.405 μm for *t_pmd(imd/aan)elec_* is less than the value of 0.6725 μm for *t_pmd(m1/aan)elec_* due to the additional etching of *t_pmd(imd/aan)elec_* during the m1 patterning.

Given the value for *t_imd(m2/pmd)elec_* in Eq. (4) we can also separate the oxides in #12 and #13 in Table 6 using the following two calculations, respectively:

$$\begin{array}{l} t_{pmd\left(imd/p1\right)elec}={\left[t_{pmd\left(imd/p1\right)}+t_{imd\left(m2/pmd\right)}\right]}_{elec}-t_{imd\left(m2/pmd\right)elec}\\ \;=1.527-0.922=0.605\mu m. \end{array}$$ (6)

and

$$\begin{array}{l} t_{pmd\left(imd/p2\right)elec}={\left[t_{pmd\left(imd/p2\right)}+t_{imd\left(m2/pmd\right)}\right]}_{elec}-t_{imd\left(m2/pmd\right)elec}\\ \;=1.500-0.922=0.578\;\mu m. \end{array}$$ (7)

As an extension of this third assumption involving the IMD oxides, it is also assumed that

$$t_{imd\left(gl/pmd\right)phys}=t_{imd\left(gl/m1\right)phys}$$ (8)

the value for which will be determined in Sec. 5.10. These thicknesses are not found using the electrical approach.

**Fig. 7:**
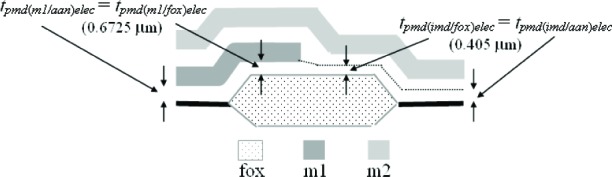
A cross section for equating some PMD thicknesses.

The fourth assumption involves the PMD oxide. Referring to Fig. 7, it is assumed that the PMD oxide thicknesses are equated as follows:

$$t_{pmd\left(m1/fox\right)elec}=t_{pmd\left(m1/ann\right)elec}=0.6725\;\mu m$$ (9)

and

$$t_{pmd\left(imd/fox\right)elec}=t_{pmd\left(imd/ann\right)elec}=0.405\;\mu m.$$ (10)

Note that *t_pmd(m1/fox)elec_* in Eq. (9) is not equated with *t_pmd(imd/fox)elec_* in Eq. (10), for example and as shown in Fig. 7, because the PMD oxide without the m1 on top undergoes an additional etch when the m1 is patterned. And, as you can see in Eqs. (9) and (10), the difference between these two values can be significant (approximately 0.268 μm in this case for the electrical approach).

Given the above equalities in Eqs. (9) and (10), the oxide thicknesses in the remaining two entries in Table 6 involving multiple oxides (namely, #6 and #10) can be separated as follows:

$$\begin{array}{l} t_{fox,m1\left(pmd/sub\right)elec}={\left[t_{fox,m1\left(pmd/sub\right)}+t_{pmd\left(m1/fox\right)}\right]}_{elec}-t_{pmd\left(m1/fox\right)}{}_{elec}\\ \;=1.3968-0.6725=0.7243\;\mu m \end{array}$$ (11)

and

$$\begin{array}{l} t_{fox,m2\left(pmd/sub\right)elec}={\left[t_{fox,m2\left(pmd/sub\right)}+t_{pmd\left(imd/fox\right)}+t_{imd\left(m2/pmd\right)}\right]}_{elec}\\ \;-t_{pmd\left(imd/fox\right)}{}_{elec}-t_{imd\left(m2/pmd\right)}{}_{elec}\\ \;=2.300-0.405-0.922=0.973\;\mu m \end{array}$$ (12)

where the added subscripts after “*fox*”, namely, “*m1*” or “*m2*”, indicate the thickness of the field oxide beneath m1 or m2, respectively. Indeed, *t_fox,m1(pmd/sub)elec_* in Eq. (11) should equal *t_fox,m2(pmd/sub)elec_* in Eq. (12), and the large discrepancy of approximately 0.249 μm between these two measurements will be discussed in Sec. 7. This discrepancy will be the starting point for obtaining an additional uncertainty component for thicknesses obtained from capacitances.

The newly found thicknesses presented in this section are included in Table 8 under the column heading *t_elec_*, for the thicknesses found using the electrical approach. For the thicknesses found using the physical approach, the column heading *t_phys_* is used. Most of these thicknesses will be obtained in Sec. 5.

## 5. Comparing Approaches

Thicknesses obtained with the physical approach can be compared with those obtained with the electrical approach. The thicknesses with the smaller combined standard uncertainty values would be the preferred thickness values, for use in Young’s modulus calculations, for example. For the comparison of these two approaches, we begin with the assumptions, which are presented in Sec. 5.1, followed by crystal lattice calculations in Sec. 5.2. Then, in Secs. 5.3 through 5.6, we compare field oxide thicknesses obtained with the physical and the electrical approach using TS #1 (shown in Fig. 1). Section 5.7 presents additional thickness comparisons that can be made given the platforms in this test structure. Then, Sec. 5.8 presents some thickness comparisons using the platforms in TS #2 (shown in Fig. 2). Using step-height measurements from this test structure, the physical polysilicon interconnect thicknesses are found in Sec. 5.9. And finally, Sec. 5.10 presents the determination of the physical metal interconnect thicknesses using both TS #2 (shown in Fig. 2) and TS #3 (shown in Fig. 3). At this point, all of the electrical thicknesses will have been compared in some manner with physical thicknesses, thereby completing the measurement comparisons before the post-processing XeF_2_ etch.

### 5.1 Assumptions

**Fig. 8:**
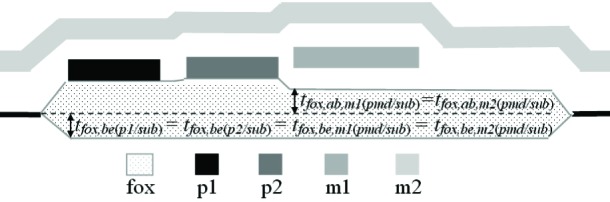
The field oxide thickness below the level of the unoxidized active area is assumed to remain the same.

As the physical approach is compared to the electrical approach, five assumptions are made. First, it is assumed that the thickness of the field oxide below the level of the unoxidized active area is the same regardless of what layers (such as p1, p2, m1, or m2) are above it. In other words, as can be seen in Fig. 8,

$$t_{fox,be\left(p1/sub\right)}=t_{fox,be\left(p2/sub\right)}=t_{fox,be,m1\left(pmd/sub\right)}=t_{fox,be,m2\left(pmd/sub\right)}$$ (13)

where the added subscripted letters “*be*” indicate the thickness is of the field oxide that is below the unoxidized active area level.

Second, the gate oxide thicknesses (i.e., #2 and #4 in Table 6) are assumed to be accurate. With capacitance measurements, the thicknesses of these thinner gate oxides are more accurately determined than the thicknesses of the thicker deposited oxides. Therefore, we will equate these measurements for the physical and electrical approaches as follows:

$$t_{thin\left(p1/aan\right)phys}=t_{thin\left(p1/aan\right)elec}=0.03130\;\mu m$$ (14)

and

$$t_{thin\left(p2/aan\right)phys}=t_{thin\left(p2/aan\right)elec}=0.04878\;\mu m.$$ (15)

To keep the physical approach somewhat independent of the electrical approach, we are going to the extreme of equating these oxides as an assumption so that we are careful in not “mixing” the approaches in the many equations that follow. In Sec. 7, this will enable us to discuss data trends between the physical approach and the electrical approach.

Third, it is assumed that #1 in Table 6 is accurate such that

$$t_{fox\left(p1/sub\right)phys}=t_{fox\left(p1/sub\right)elec}=0.8846\;\mu m.$$ (16)

This assumption is used in Sec. 5.2.

The fourth assumption is dependent upon the *u_c_* values in the given data set, therefore, the specific details associated with this assumption will be presented in Secs. 5.7 and 5.8. The upshot is that for the presented data set, we will assume that #8, #11, and #14 in Table 6 are accurate. Therefore, we assume the following equalities:

$$t_{pmd\left(m1/p1\right)phys}=t_{pmd\left(m1/p1\right)elec}=0.7516\;\mu m,$$ (17)

$$\begin{array}{l} {\left[t_{pmd\left(imd/aan\right)}+t_{imd\left(m2/pmd\right)}\right]}_{phys}=\\ {\left[t_{pmd\left(imd/aan\right)}+t_{imd\left(m2/pmd\right)}\right]}_{elec}=1.327\mu m, \end{array}$$ (18)

and

$$t_{imd\left(m2/m1\right)phys}=t_{imd\left(m2/m1\right)elec}=0.922\mu m.$$ (19)

And fifth, we will equate the same similar oxides for the physical approach as we did in Sec. 4.3 for the electrical approach. In other words, similarly to Eqs. (4), (9), and (10), we have the following:

$$t_{imd\left(m2/pmd\right)phys}=t_{imd\left(m2/m1\right)phys}=0.922\mu m,$$ (20)

$$t_{pmd\left(m1/fox\right)phys}=t_{pmd\left(m1/aan\right)phys},$$ (21)

and

$$t_{pmd\left(imd/fox\right)phys}=t_{pmd\left(imd/aan\right)phys}.$$ (22)

The values for Eqs. (21) and (22) will be determined in Secs. 5.8 and 5.7, respectively.

Table 9 includes a listing of most of the equated oxides in this paper, including the ones presented up to this point.

### 5.2 Crystal Lattice Calculations

Crystal lattice calculations are used to determine the amount of field oxide above and below the unoxidized active area level. For the electrical approach, the relative volumes of silicon dioxide product and silicon reactant can be predicted [9,14], keeping in mind that for a planar process, the length and width are constant and only the thicknesses change. We have the following calculation:

$$\begin{array}{l} \frac{t_{Si}}{t_{SiO_{2}}}=\frac{V_{molar,Si}}{V_{molar,SiO_{2}}}=\frac{\left(w_{Si}/\rho_{Si}\right)}{\left(w_{SiO_{2}}/\rho_{SiO_{2}}\right)}\\ \;=\frac{\left(28.1g/\mathrm{mol}\right)/\left(2.33{g/\mathrm{cm}}^{3}\right)}{\left(60.1g/\mathrm{mol}\right)/\left(2.21{g/\mathrm{cm}}^{3}\right)}=0.443 \end{array}$$ (23)

where *Vmolar* is the molar volume of silicon (*Si*) or silicon dioxide (*SiO*_2_) as indicated by the subscript, *w* is the molecular weight, and *ρ* is the density. This means that the thickness of the original silicon, which has been converted now to silicon dioxide, is 44.3 % of the final total oxide thickness. In other words, the field oxide should extend 44.3 % below and 55.7 % above the unoxidized active area level. This is for the ideal case and actual processes may be biased away from the ideal; therefore, the one sigma uncertainty for each of these percentages will be assumed to be 1.5 %.

In addition to field oxide, the above calculation is also applicable to the three thin, thermal oxides given in Table 4, namely, *t_thin(p1/aan)_*, *t_thin(p2/aan)_*, and *t_thin(p2/p1)_*. In referring to the oxide thickness above the unoxidized active area level, the added subscripted letters “*ab*” are added after the specified oxide, as in *t_fox,ab(p2/sub)_* and *t_thin,ab(p1/aan)_*. Similarly, in referring to the oxide thickness below the unoxidized active area level, the subscripted letters “*be*” are used, as in *t_fox,be(p1/sub)_* and *t_thin,be(p2/aan)_*. When the subscripts “*ab*” and “*be*” are used with *t_thin(p2/p1)_*, the level of the unoxidized p1 is the reference point.^8^

**Fig. 9:**
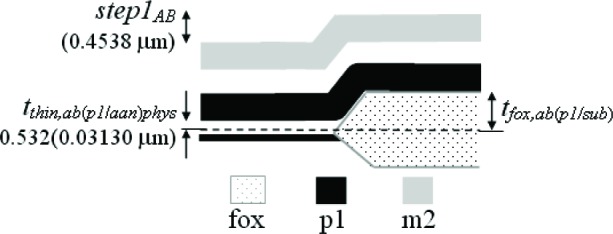
Cross section of *step1_AB_* of thickness TS #1.

To determine the percentages for the physical approach, let us take a close look at *step1_AB_* in Fig. 1b, as magnified in Fig. 9.

Given the underlying assumption in #7 in Table 9, we can write the following step-height equation:

$$\begin{array}{l} t_{fox,ab\left(p1/sub\right)phys}=step1_{AB}+t_{thin,ab\left(p1/aan\right)phys}\\ \;=0.4538+\frac{\%t_{ab,phys}}{100\%}\left[t_{thin\left(p1/aan\right)phys}\right]\\ \;=0.4538+\frac{\%t_{ab,phys}}{100\%}\left(0.03130\right) \end{array}$$ (24)

where here %*t_ab,phys_* is the percentage of oxide above the unoxidized active area level for the physical approach. (The values for *step1_AB_* and *t_thin(p1/aan)phys_* can be found in Tables 5 and 9, respectively.)

Therefore, for the physical approach, the percentage of oxide above (%*t_ab,phys_*) and the percentage of oxide below (%*t_be,phys_*) the unoxidized active area level can be calculated as follows:

$$\begin{array}{c} \%t_{ab,phys}=\frac{t_{fox,ab\left(p1/sub\right)phys}}{t_{fox\left(p1/sub\right)phys}}\times 100\%\\ =\frac{0.4538+\frac{\%t_{ab,phys}}{100\%}\left(0.03130\right)}{0.8846}\times 100\%=53.2\% \end{array}$$ (25)

and

$$\%t_{be,phys}=100\%-\%t_{ab,phys}=46.8\%.$$ (26)

The one sigma uncertainty for each of these percentages will be assumed to be 1.5 %.

Due to the relatively small dimensions associated with the conversion of the silicon or polysilicon into a thin, thermal oxide, in some figures, this small reduction in thickness may not be indicated. However, it needs to be accounted for during pertinent calculations.

### 5.3 Examining *Step1_AB_*

First, let us reexamine *step1_AB_* in Fig. 1b, as magnified in Fig. 9. We can use calculations from this step for comparison with similar values derived from #1 in Table 6. Since we have a value for %*t_ab,phys_*, we can now complete the calculation for *t_fox,ab(p1/sub)phys_* in Eq. (24) as follows:

$$\begin{array}{l} t_{fox,ab\left(p1/sub\right)phys}=step1_{AB}+t_{thin,ab\left(p1/aan\right)phys}\\ \;=0.4538+\frac{\%t_{ab,phys}}{100\%}\left[t_{thin\left(p1/aan\right)phys}\right]\\ \;=0.4538+\frac{\%t_{ab,phys}}{100\%}\left(0.03130\right)\\ \;=0.4538+\frac{53.2}{100}\left(0.03130\right)\\ \;=0.4705\mu m. \end{array}$$ (27)

And, by definition and using the underlying assumption in #9 in Table 9, we can write the following:

$$\begin{array}{l} t_{fox,be\left(p1/sub\right)phys}=t_{fox\left(p1/sub\right)phys}-t_{fox,ab\left(p1/sub\right)phys}\\ \;=0.8846-0.4705=0.4141\;\mu m. \end{array}$$ (28)

For the electrical approach, we can use the following equations to compare with Eqs. (27) and (28):

$$\begin{array}{l} t_{fox,ab\left(p1/sub\right)elec}=\left(0.557\right)t_{fox\left(p1/sub\right)elec}\\ \;=\left(0.557\right)\left(0.8846\right)=0.4927\;\mu m \end{array}$$ (29)

and

$$\begin{array}{l} t_{fox,be\left(p1/sub\right)elec}=\left(0.443\right)t_{fox\left(p1/sub\right)elec}\\ \;=\left(0.443\right)\left(0.8846\right)=0.3919\;\mu m. \end{array}$$ (30)

Due to the difference of the calculated values in Eqs. (27) and (29) (namely, 0.0222 μm) the physical and the electrical approaches give fairly comparable results for the amount of field oxide above and below the unoxidized active area level. Several different tacks can be used to find the physical field oxide thicknesses. The one presented here, of equating *t_fox(p1/sub)phys_* with *t_fox(p1/sub)elec_*, offers the advantage of comparing the percentages. The above results are included in Table 10. Section 7 will provide a further discussion of these results. Consult the appendix for the equation used to determine the combined standard uncertainty values.

### 5.4 Examining *Step1_CD_*

**Fig. 10:**
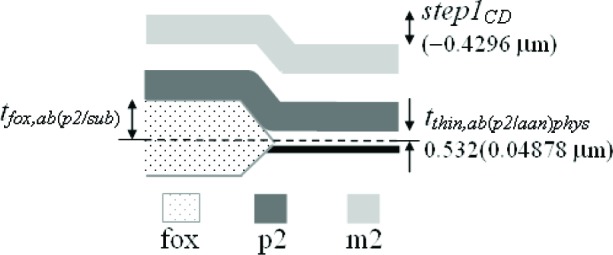
Cross section of *step1_CD_* of thickness TS #1.

Next, let us look at *step1_CD_* in Fig. 10. We can use calculations from this step for comparison with similar values derived from or using #3 in Table 6 for the electrical approach.

Given the underlying assumption in #8 in Table 9, we can make the following step-height calculation:

$$\begin{array}{l} t_{fox,ab\left(p2/sub\right)phys}=-step1_{CD}+t_{thin,ab\left(p2/aan\right)phys}\\ \;=0.4296+0.532\left[t_{thin\left(p2/aan\right)phys}\right]\\ \;=0.4296+0.532\left(0.04878\right)\\ \;=0.4556\;\mu m. \end{array}$$ (31)

from which we can calculate *t_fox(p2/sub)phys_* to be:

$$\begin{array}{l} t_{fox\left(p2/sub\right)phys}=t_{fox,ab\left(p2/sub\right)phys}+t_{fox,be\left(p2/sub\right)phys}\\ \;=0.4556+0.4141=0.8697\;\mu m \end{array}$$ (32)

recalling the underlying assumption in #6a in Table 9 equating *t_fox,be(p2/sub)phys_* with *t_fox,be(p1/sub)phys_*.

For the electrical approach, using the underlying assumption in #5a in Table 9, we have the following:

$$\begin{array}{l} t_{fox,ab\left(p2/sub\right)elec}=t_{fox\left(p2/sub\right)elec}-t_{fox,be\left(p2/sub\right)elec}\\ \;=0.8892-0.3919=0.4973\;\mu m. \end{array}$$ (33)

The above two measurements in Eqs. (31) and (33) for *t_fox,ab(p2/sub)_* differ by 0.0417 μm. These measurements are included in Table 10. Section 7 will provide a further discussion of these results. Also, the two measurements for *t_fox(p2/sub)_* in #2 of Table 8 differ by 0.0195 μm and the electrical measurement is preferred due to the lower value for *u_c_*.

### 5.5 Examining *Step1_EF_*

**Fig. 11:**
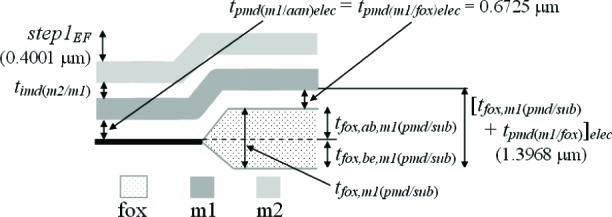
Cross section of *step1_EF_* of thickness TS #1.

Next, let us look at *step1_EF_* in Fig. 11. This step-height measurement will be compared with a similar value derived from #6 in Table 6. We can make the following step-height calculation:

$$t_{fox,ab,m1\left(pmd/sub\right)phys}=step1_{EF}=0.4001\;\mu m$$ (34)

from which we can calculate *t_fox,m1(pmd/sub)phys_* to be:

$$\begin{array}{l} t_{fox,m1\left(pmd/sub\right)phys}=t_{fox,ab,m1\left(pmd/sub\right)phys}\\ \;+t_{fox,be,m1\left(pmd/sub\right)phys}\\ \;=0.4001+0.4141=0.8142\;\mu m \end{array}$$ (35)

with the help of the underlying assumption in #6b in Table 9.

For the electrical approach, using the underlying assumptions in #3a and #5b in Table 9, we have the following:

$$\begin{array}{l} t_{fox,ab,m1\left(pmd/sub\right)elec}={\left[t_{fox,m1\left(pmd/sub\right)}+t_{pmd\left(m1/fox\right)}\right]}_{elec}\\ \;-t_{pmd\left(m1/fox\right)elec}\\ \;-t_{fox,be,m1\left(pmd/sub\right)elec}\\ \;=1.3968-0.6725-0.3919\\ \;=0.3324\;\mu m. \end{array}$$ (36)

The above calculations for *t_fox,ab,m1(pmd/sub)_* in Eqs. (34) and (36) are given in Table 10. They differ by 0.0677 μm. Section 7 will provide a further discussion of these results. Also, the two measurements for *t_fox,m1(pmd/sub)_* in #3a in Table 8 differ by 0.0899 μm and the electrical measurement is preferred due to the lower value of *u_c_*.

### 5.6 Examining *Step1_GH_*

**Fig. 12:**
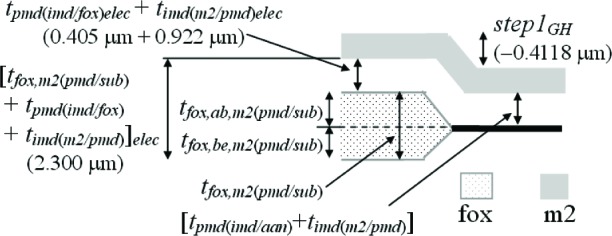
Cross section of *step1_GH_* of thickness TS #1.

Next, let us look at *step1_GH_* in Fig. 12. This step-height measurement will be compared with a similar value derived from #10 in Table 6 for the electrical approach. We can make the following step-height calculation:

$$t_{fox,ab,m2\left(pmd/sub\right)phys}=-step1_{GH}=0.4118\;\mu m$$ (37)

from which we can calculate *t_fox,m2(pmd/sub)phys_* to be:

$$\begin{array}{l} t_{fox,m2\left(pmd/sub\right)phys}=t_{fox,ab,m2\left(pmd/sub\right)phys}\\ \;+t_{fox,be,m2\left(pmd/sub\right)phys}\\ \;=0.4118+0.4141\\ \;=0.8259\;\mu m, \end{array}$$ (38)

with the help of the underlying assumption in #6c in Table 9.

For the electrical approach, using the underlying assumptions in #1a, #4a, and #5c in Table 9, we have the following:

$$\begin{array}{l} t_{fox,ab,m2\left(pmd/sub\right)elec}=[t_{fox,m2\left(pmd/sub\right)}+t_{pmd\left(imd/fox\right)}\\ \;{+t_{imd\left(m2/pmd\right)}]}_{elec}\\ \;-t_{fox,be,m2\left(pmd/sub\right)elec}\\ \;-t_{pmd\left(imd/fox\right)elec}\\ \;-t_{imd\left(m2/pmd\right)elec}\\ \;=2.300-0.3919-0.405\\ \;-0.922\\ \;=0.581\;\mu m. \end{array}$$ (39)

The above calculations for *t_fox,ab,m2(pmd/sub)_* in Eqs. (37) and (39) are given in Table 10. They differ by approximately 0.169 μm. Section 7 will provide a further discussion of these results. Also, the two measurements for *t_fox,m2(pmd/sub)_* in #3b in Table 8 differ by approximately 0.147 μm and the physical measurement is preferred due to the lower value of *u_c_*.

### 5.7 Additional Comparisons Using TS #1

**Fig. 13:**
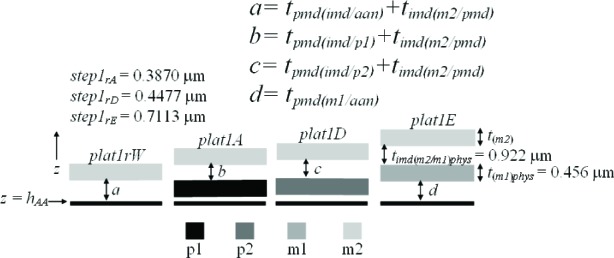
Cross section of various platforms of thickness TS #1.

Additional thickness comparisons can be made using TS #1. Looking at Table 6, we have already addressed #1, #3, #6, and #10 by way of field oxide thickness comparisons in the previous four sections. In this section, we will compare calculations from or results in #11, #12, #13, and #7 with calculations using or values for *a*, *b*, *c*, and *d*, respectively, in Fig. 13. (Figure 13 consists of select platforms from TS #1.) For the following calculations, we can assume that *t_(m2)_* = 0 since m2 is common to each platform.

In Sec. 5.1, we assumed in the fourth assumption that #11 in Table 6 is accurate. Why did we assume this? To make the calculations for *a*, *b*, *c*, and *d* in Fig. 13, the height of the unoxidized active area, *h_AA_*, can be used as a reference level. Therefore, we choose the approach that produces the lowest value of *u_c_* for *h_AA_*. The layer combinations to be considered are #41, #42, #43, and #45 in Table 4, which have m2 as the top layer and one or fewer interconnects beneath the m2 layer.^9^ The equation used to obtain *u_c_* is presented in the appendix. Suffice it to say that after calculating the values for *u_c_* for each layer combination for this data set, #41 produces the smallest value. (The oxide in #41 corresponds to #11 in Table 6.) Therefore, we can obtain the smallest *u_c_* value for *h_AA_* if we assume the following:

$$\begin{array}{l} a={\left[t_{pmd\left(imd/aan\right)}+t_{imd\left(m2/pmd\right)}\right]}_{phys}\\ \;={\left[t_{pmd\left(imd/aan\right)}+t_{imd\left(m2/pmd\right)}\right]}_{elec}\\ \;=1.327\;\mu m \end{array}$$ (40)

as given in Eq. (18) and use *a* in as many of the remaining calculations as possible.

The appendix points out that step-height measurements are preferred over platform-height measurements. Therefore, we can calculate *b* for the physical approach (which corresponds to #12 in Table 6 for the electrical approach) using *step1_rA_* as follows:

$$\begin{array}{l} \;a+step1_{rA}=t_{thin,ab\left(p1/aan\right)phys}+t_{\left(p1\right)elec}+b\\ 1.327+0.3870=0.532\left(0.03130\right)+0.3102+b\\ \;b={\left[t_{pmd\left(imd/p1\right)}+t_{imd\left(m2/pmd\right)}\right]}_{phys}\\ \;b=1.387\mu m \end{array}$$ (41)

and we can calculate *c* for the physical approach (which corresponds to #13 in Table 6 for the electrical approach) using *step1_rD_* as follows:

$$\begin{array}{l} \;a+step1_{rD}=t_{thin,ab\left(p2/aan\right)phys}+t_{\left(p2\right)elec}+c\\ 1.327+0.4477=0.532\left(0.04878\right)+0.3571+c\\ \;c={\left[t_{pmd\left(imd/p2\right)}+t_{imd\left(m2/pmd\right)}\right]}_{phys}\\ \;c=1.392\;\mu m. \end{array}$$ (42)

In the above calculations for *b* and *c*, we used the electrical thicknesses for p1 and p2. We will see in Sec. 5.9 that the *u_c_* values for the electrical poly interconnect thicknesses are less than the *u_c_* values for the physical poly interconnect thicknesses.

It was also assumed in the fourth assumption in Sec. 5.1 [see Eq. (19)] that #14 in Table 6 is accurate. Why? Given the underlying assumption in #1c in Table 9 for which *t_imd(m2/pmd)phys_* is common to Eqs. (40), (41), and (42), we can separate the PMD and IMD oxides in *a*, *b*, and *c* in Eqs. (40), (41), and (42). Therefore, assuming #14 in Table 6 to be accurate, gives us a low value for *u_c_* for the calculations that follow:

$$\begin{array}{l} t_{pmd\left(imd/aan\right)phys}={\left[t_{pmd\left(imd/aan\right)}+t_{imd\left(m2/pmd\right)}\right]}_{phys}\\ \;-t_{imd\left(m2/pmd\right)phys}\\ \;=a-t_{imd\left(m2/pmd\right)phys}\\ \;=1.327-0.922\\ \;=0.405\;\mu m, \end{array}$$ (43)

$$\begin{array}{l} t_{pmd\left(imd/p1\right)phys}={\left[t_{pmd\left(imd/p1\right)}+t_{imd\left(m2/pmd\right)}\right]}_{phys}\\ \;-t_{imd\left(m2/pmd\right)phys}\\ \;=b-t_{imd\left(m2/pmd\right)phys}\\ \;=1.387-0.922\\ \;=0.465\;\mu m, \end{array}$$ (44)

and

$$\begin{array}{l} t_{pmd\left(imd/p2\right)phys}={\left[t_{pmd\left(imd/p2\right)}+t_{imd\left(m2/pmd\right)}\right]}_{phys}\\ \;-t_{imd\left(m2/pmd\right)phys}\\ \;=c-t_{imd\left(m2/pmd\right)phys}\\ \;=1.392-0.922\\ \;=0.470\;\mu m. \end{array}$$ (45)

Given the underlying assumption in #4b in Table 9, we now have a value for this equality, namely:

$$t_{pmd\left(imd/fox\right)phys}=t_{pmd\left(imd/aan\right)phys}=0.405\;\mu m.$$ (46)

The measurements in Eqs. (43), (44), and (45) are presented in Table 8 as #11, #13, and #14, respectively, for comparison with the corresponding electrical thicknesses.

Additionally, given *step1_rE_* in Fig. 13, we can calculate *d* as follows:

$$\begin{array}{l} \;a+step1_{rE}=d+t_{\left(m1\right)phys}+t_{imd\left(m2/m1\right)phys}\\ 1.327+0.7113=d+0.456+0.922\\ \;d=t_{pmd\left(m1/aan\right)phys}=0.660\;\mu m. \end{array}$$ (47)

For this calculation, we use the physical thickness of m1, as we will see in Sec. 5.10 that *u_c_* for *t_(m1)phys_* is less than *u_c_* for *t_(m1)elec_*. (An alternate calculation of *d* will be found in the next section, which ends up having a smaller value for *u_c_*.)

### 5.8 Some Thickness Comparisons Using TS #2

**Fig. 14:**
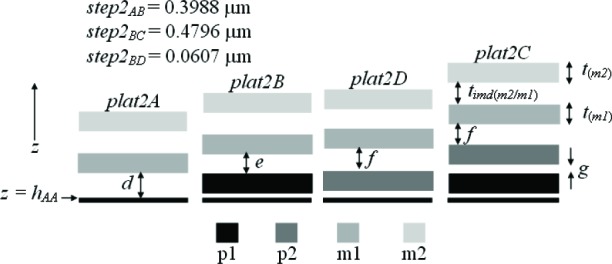
Cross section of various platforms of thickness TS #2.

In this section, additional thickness comparisons will be made. We will compare #5, #7, #8, and #9 in Table 6 with the results from step-height calculations for *g*, *d*, *e*, and *f*, respectively, as depicted in Fig. 14, which shows select platforms from TS #2. For the calculations associated with this figure, we will assume that *t_(m2)_* = 0, *t_imd(m2/m1)_* = 0, and *t_(m1)_* = 0 because the corresponding layers are common to each platform in this figure and therefore their values would cancel out in the calculations.

As done in the previous section, we first find the lowest value of *u_c_* for *h_AA_*. The layer combinations to be considered are #45 to #48 in Table 4, which have m2 as the top layer and which include m1 as an interconnect. After calculating the values for *u_c_* for each layer combination for this data set, #46 produces the smallest value,^10^ so we will assume Eq. (17) and use *e* equals 0.7516 μm in as many calculations as possible. Therefore, given the value for *e*, we can calculate *d*, *f*, and *g* in Fig. 14. Refer to Table 8 for the appropriate values to insert in the equations that follow:

$$\begin{array}{c} d+step2_{AB}=t_{thin,ab\left(p1/aan\right)phys}+t_{\left(p1\right)elec}+e\\ d+0.3988=0.532\left(0.03130\right)+0.3102+0.7516\\ d=t_{pmd\left(m1/aan\right)phys}=0.6797\;\mu m, \end{array}$$ (48)

$$\begin{array}{c} t_{thin,ab\left(p1/aan\right)phys}+t_{\left(p1\right)elec}+e+step2_{BD}\\ =t_{thin,ab\left(p2/aan\right)phys}+t_{\left(p2\right)elec}+f\\ 0.532\left(0.03130\right)+0.3102+0.7516+0.0607\\ =0.532\left(0.04878\right)+0.3571+f\\ f=t_{pmd\left(m1/p2\right)phys}=0.7561\;\mu m, \end{array}$$ (49)

and

$$\begin{array}{c} e+step2_{BC}=g_{ab}+t_{\left(p2\right)elec}+f\\ 0.7516+0.4769=0.532g+0.3571+0.7561\\ g=t_{thin\left(p2/p1\right)phys}=0.222\;\mu m. \end{array}$$ (50)

Once again, *t_(p1)elec_* and *t_(p2)elec_* were used instead of *t_(p1)phys_* and *t_(p2)phys_*, respectively. These values for *d*, *e*, *f*, and *g* are presented in Table 8 as #7b, #9, #10, and #6, respectively, for comparison with the corresponding electrical thicknesses. We will use #7b in Table 8 as the preferred physical calculation for *d* due to the lower value for *u_c_* as opposed to #7a.

### 5.9 Polysilicon Interconnect Thicknesses

In this section, we will determine the physical p1 and p2 interconnect thicknesses for comparison with similar values obtained with the electrical approach in Sec. 4.2. We will also determine the reduced p1 thicknesses *t_(p1_*_′_*_)phys_* and *t_(p1_*_′_*_)elec_*.

**Fig. 15:**
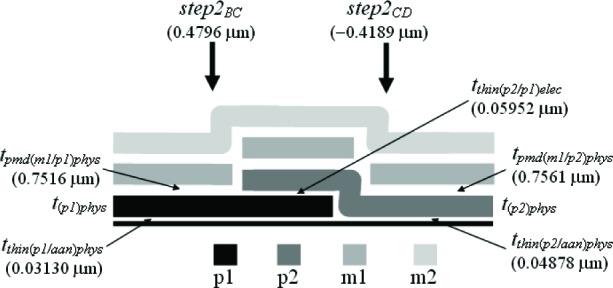
Cross section of a portion of thickness TS #2.

Referring to Tables 5 and 8, the reduced p1 thickness, namely *t_(p1_*_′_*_)phys_*, is calculated from *step2_CD_* in Fig. 15 as follows:

$$\begin{array}{l} -step2_{CD}+t_{thin,ab\left(p2/aan\right)phys}\\ \;=t_{thin,ab\left(p1/aan\right)phys}+t_{\left(p1^{\prime }\right)phys}+t_{thin\left(p2/p1\right)elec}\\ 0.4189+0.532\left(0.04878\right)\\ \;=0.532\left(0.03130\right)+t_{\left(p1^{\prime }\right)phys}+0.05952\\ t_{\left(p1^{\prime }\right)phys}=0.3687\;\mu m \end{array}$$ (51)

from which *t_(p1)phys_* is calculated as follows:

$$\begin{array}{l} t_{\left(p1\right)phys}=t_{\left(p1^{\prime }\right)phys}+0.468\left[t_{thin\left(p2/p1\right)elec}\right]\\ \;=0.3687+0.468\left(0.05952\right)\\ \;=0.3966\;\mu m \end{array}$$ (52)

where *t_thin(p2/p1)elec_* is used in conjunction with the physical percentage instead of *t_thin(p2/p1)phys_* in conjunction with the physical percentage.^11^ The above p1 physical thicknesses are included in Table 8 along with the corresponding electrical thicknesses, including the reduced p1 electrical thickness calculated as follows:

$$\begin{array}{l} t_{\left(p1^{\prime }\right)elec}=t_{\left(p1\right)elec}-0.443\left[t_{thin\left(p2/p1\right)elec}\right]\\ \;=0.3102-0.443\left(0.05952\right)\\ \;=0.2838\;\mu m. \end{array}$$ (53)

Similarly, the p2 thickness can be determined from *step2_BC_* in Fig. 15 using the equation:

$$\begin{array}{l} step2_{BC}+t_{pmd\left(m1/p1\right)phys}\\ \;=0.532\left[t_{thin\left(p2/p1\right)elec}\right]+t_{\left(p2\right)phys}+t_{pmd\left(m1/p2\right)phys}\\ 0.4796+0.7516\\ \;=0.532\left(0.05952\right)+t_{\left(p2\right)phys}+0.7561\\ t_{\left(p2\right)phys}=0.4434\;\mu m. \end{array}$$ (54)

Once again, note that *t_thin(p2/p1)elec_* was used in conjunction with the physical percentage instead of *t_thin(p2/p1)phys_* with the physical percentage due to the large discrepancies in the corresponding values for *u_c_* as given in #6 in Table 8 in conjunction with the fact that this thickness is so small that the electrical approach can be assumed to be accurate. This was not assumed earlier since we had an opportunity to calculate it using the physical approach.

Looking at #19, #20, and #21 in Table 8, the electrical approach yields the more trusted values for *t_(p1)_*, *t_(p1_*_′_*_)_*, and *t_(p2)_*, respectively.

### 5.10 Metal Interconnect Thicknesses

In this section, we will determine the physical m1 and m2 interconnect thicknesses for comparison with similar values obtained with the electrical approach in Sec. 4.2.

**Fig. 16:**
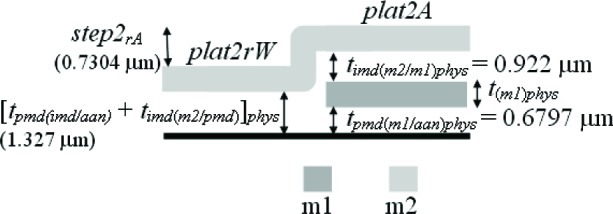
Cross section of two platforms in thickness TS #2 to determine the physical m1 thickness.

To determine the m1 physical thickness, refer to Fig. 16, which uses select platforms from TS #2. We can make the following calculation with the help of Table 8 and the underlying assumption in #4d in Table 9:

$$\begin{array}{l} {\left[t_{pmd\left(imd/aan\right)}+t_{imd\left(m2/pmd\right)}\right]}_{phys}+step2_{rA}\\ \;=t_{pmd\left(m1/aan\right)phys}+t_{\left(m1\right)phys}+t_{imd\left(m2/m1\right)phys}\\ 1.327+0.7340=0.6797+t_{\left(m1\right)phys}+0.922\\ t_{\left(m1\right)phys}=0.456\;\mu m. \end{array}$$ (55)

As seen in Table 8, *t**_(m1)phys_* is preferred over *t_(m1)elec_*.

**Fig. 17:**
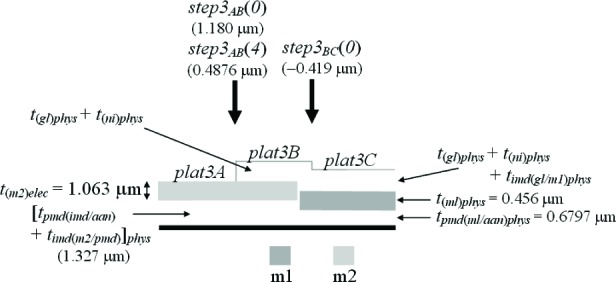
Cross section of a portion of thickness TS #3. For interferometric measurements, TS #3 must be topped with a smooth reflective layer, such as gold. Chromium is typically used to help the gold adhere to the chip.

Before the m2 physical thickness can be found, #17 and #18 in Table 8, which are assumed to be equal according to the underlying assumption in #2 in Table 9, should be obtained. These thicknesses cannot be found with the electrical approach. They can be found from *step3_BC_(0)* in TS #3 (see Fig. 17). Since the top layer is not reflective, for interferometric measurements, additional post-processing is required (see Sec. 2.2). The following equations can be written assuming that the glass and nitride cap thicknesses do not vary with topography:

$$\begin{array}{l} t_{imd\left(gl/m1\right)phys}={\left[t_{pmd\left(imd/aan\right)}+t_{imd\left(m2/pmd\right)}\right]}_{phys}\\ \;+t_{\left(m2\right)elec}+step3_{BC}\left(0\right)\\ \;-t_{pmd\left(m1/aan\right)phys}-t_{\left(m1\right)phys}\\ \;=a+t_{\left(m2\right)elec}+step3_{BC}\left(0\right)\\ \;-t_{pmd\left(m1/aan\right)phys}-t_{\left(m1\right)phys}\\ \;=1.327+1.063-0.419\\ \;-0.6797-0.456\\ \;=0.835\;\mu m \end{array}$$ (56)

and

$$t_{imd\left(gl/pmd\right)phys}=t_{imd\left(gl/m1\right)phys}=0.835\;\mu m.$$ (57)

**Fig. 18:**
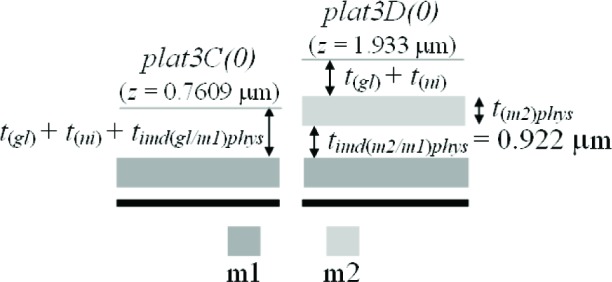
Cross section of two platforms in thickness TS #3 to determine the physical m2 thickness. For interferometric measurements, TS #3 must be topped with a smooth reflective layer, such as gold. Chromium is typically used to help the gold adhere to the chip.

To determine the m2 physical thickness, refer to Fig. 18, which also uses select platforms from TS #3. Once again the post-processing mentioned in Sec. 2.2 is needed for interferometric measurements since the top layer is not reflective. We can make the following calculation:^12^

$$\begin{array}{l} plat3D\left(0\right)-plat3C\left(0\right)\\ \;=t_{imd\left(m2/m1\right)phys}+t_{\left(m2\right)phys}-t_{imd\left(gl/m1\right)phys}\\ 1.933-0.7609=0.922+t_{\left(m2\right)phys}-0.835\\ \;t_{\left(m2\right)phys}=1.085\;\mu m. \end{array}$$ (58)

Since *t_(m2)elec_* was used to find *t_imd(gl/m1)phys_*, which is used in the calculation of *t_(m2)phys_*, we cannot expect the *u_c_* value for *t_(m2)phys_* to be less than it is for *t_(m2)elec_*, as confirmed by the results in #23 of Table 8.

## 6. Thickness Calculations Remaining After the Post-Processing Etch

The remaining thicknesses to be found are #24 and #25 in Table 8. This analysis assumes that the m2 thickness is not reduced during the glass etch and that the m2, glass, and nitride cap (assuming it remains) thicknesses are not reduced during the XeF_2_ etch.

Let’s consider two separate measurements for *step3_AB_* in Fig. 17. First, let’s look at the pre-XeF_2_ etch measurement of *step3_AB_* [or *step3_AB_(0)*]. For this measurement, the nitride cap layer is present on top of the glass layer covering the m2 in *plat3B*. We have the following formula:

$$t_{\left(gl\right)phys}+t_{\left(ni\right)phys}=Step3_{AB}\left(0\right)=1.180\;\mu m.$$ (59)

With Eq. (59) alone, the glass layer and the nitride cap layer are not separated. As a rough approximation we can separate these layers with the use of the following equation:

$$t_{\left(gl\right)phys}\approx t_{\left(ni\right)phys}.$$ (60)

However, to be more exact, especially if we are interested in the CMOS thicknesses after a XeF_2_ etch, all of the previously obtained thicknesses still apply; however, we would also need an additional measurement of *step3_AB_* after the etch. After the etch, if the nitride cap is still present on top of the glass layer covering the m2 on *plat3B*, *step3_AB_* is called *step3_AB_(n)*^+^, where *n* indicates the number of XeF_2_ etch cycles and the “+” sign indicates the nitride cap layer is still present. If the nitride cap is no longer present, *step3_AB_* is called s*tep3_AB_(n)*^−^, where the “−” sign indicates the nitride cap layer is no longer present. In the former case, we would have an equation similar to Eq. (59), namely:

$$t_{\left(gl\right)phys}+t_{\left(ni\right)phys}=step3_{AB}{\left(n\right)}^{+}.$$ (61)

And, in the later case, we would have:

$$t_{\left(gl\right)phys}=step3_{AB}{\left(n\right)}^{-}.$$ (62)

When the chips are received from MOSIS, the combined glass and nitride cap thickness is approximately 1.0 μm (in this case 1.180 μm), and the glass thickness is approximately equal to the nitride cap thickness. Therefore, if the measurement of *step3_AB_* after the XeF_2_ etch is closer to 0.5 μm than it is to 1.0 μm as it is in this case [i.e., *step3_AB_(4)* = 0.4876 μm], it can be assumed that the nitride cap has lifted off, thereby enabling the calculation of the glass thickness using Eq. (62), such that

$$t_{\left(gl\right)phys}=step3_{AB}{\left(4\right)}^{-}=0.4876\;\mu m$$ (63)

followed by the calculation of the nitride cap thickness using Eq. (59), such that

$$\begin{array}{l} t_{\left(ni\right)phys}=step3_{AB}\left(0\right)-t_{\left(gl\right)phys}\\ \;=1.180-0.4876=0.692\;\mu m. \end{array}$$ (64)

## 7. Discussion

In this section, we will start in Sec. 7.1 and determine if there are any noticeable trends in the data concerning thicknesses obtained with the physical approach and thicknesses obtained with the electrical approach. In Sec. 7.2, we will discuss the variations in the field oxide thicknesses given in Table 10. Next, we will look for inconsistencies in the data, in Sec. 7.3, followed in Sec. 7.4 by a determination of whether or not a more detailed error analysis is required. Then, in Sec. 7.5, an uncertainty component will be added to the physical approach and to the electrical approach after which the preferred thicknesses are determined.

### 7.1 Data Trends

For the first discussion point, we will determine if there are any noticeable trends in the data concerning thicknesses obtained with the physical approach and thicknesses obtained with the electrical approach. Forty-two thickness values were obtained. Thirty-one of these values are given in Table 11. The remaining eleven thicknesses derived in this paper can be viewed as virtual oxide thicknesses. The subscript “*ab*” or “*be*” can be found in the designations for these oxides, which are given in Table 12. (Recall that the subscript “*ab*” indicates an oxide thickness above the unoxidized active area or unoxidized p1 level and the subscript “*be*” indicates an oxide thickness below the unoxidized active area or unoxidized p1 level.)

Look at Table 11, which is the rank-ordering of the thicknesses from smallest to largest *u_c_* value. The highlighting indicates the preferred thickness value, as determined by the lower value of *u_c_*. These are the thicknesses that would be used in Young’s modulus calculations, for example. Most (but not all) of the data in the table support the observation that the electrical approach is preferred over the physical approach for thicknesses with values of *u_c_* less than or equal to 0.011 μm. These are mostly layers that are fabricated earlier in the processing sequence, such as the poly2-to-poly1 oxide and the poly2 layers. In addition, most of the data support the conclusion that the physical approach is preferred for thicknesses with values of *u_c_* greater than or equal to 0.014 μm. These are mostly layers that are fabricated later in the processing sequence, such as the metal1 and the nitride cap. For thicknesses with *u_c_* values between 0.011 μm and 0.014 μm, inclusive, it is not clear which approach to use in further calculations; it depends on the particular thickness. Table 13 is a rank-ordering of the thicknesses from smallest to largest. No distinctive pattern is apparent from this data.

Next look at Table 14, where the capacitance values from Table 6 are rank-ordered from the largest to the smallest capacitance value. In addition to the electrical thicknesses, the physical thicknesses and their *u_c_* values are also included. The entries with the smaller *u_c_* value are highlighted, which indicates they are the preferred thickness values. This table tells us that for capacitances less than or equal to 23 aF/μm^2^ (corresponding to #12, #13, and #14) the physical approach is preferred and for larger capacitances the electrical approach is preferred. In addition, this table supports the results in Table 11 in that #12, #13, and #14 also have the largest *u_c_* values. It is not surprising that the electrical approach dominates for the thinner oxides represented by the higher capacitances in Table 14 and that the physical approach dominates for the thicker oxides because the capacitance signal becomes smaller as the oxide thickness increases.

### 7.2 Variations in Field Oxide Thickness

For the second discussion point, we will discuss the variations in the field oxide thicknesses given in Table 10, where the physical approach has *u_c_* values that are lower than the *u_c_* values for the electrical approach for all entries, and hence the physical thicknesses are the preferred thicknesses for use in calculations.

The results for the amount of field oxide above the unoxidized active area level as determined with the physical and the electrical approaches are presented in #2 through #5. In particular, note entries #4 and #5 for *t_fox,ab(pmd/sub)_*. As can be seen in Fig. 8, this thickness should be the same whether or not it is covered with m1 or m2 due to similar processing before the m1 deposition, and the physical measurements in #4 and #5 differ by 0.0117 μm whereas the electrical measurements in #4 and #5 differ by approximately 0.249 μm. (This will be discussed in greater detail in Sec. 7.5.2.) This suggests that the physical approach using step-height measurements are more reliable for one or both of these measurements. Consistent with this observation, the *u_c_* values for the physical approach are smaller than the *u_c_* values for the electrical approach for both these measurements.

Table 10 also shows a trend. For the physical approach, going from entry #2 to #3 to #4 to #5 the thickness of the field oxide above the unoxidized active area level can be viewed as getting smaller then leveling out. There is no apparent trend for the thicknesses obtained from the electrical approach. It does not seem realistic that the field oxide thickness in #5 is about 1.7 times the thickness as that given in #4 when they should be equal. Again, the *u_c_* values for the physical approach are less than the *u_c_* values for the electrical approach for these measurements.

### 7.3 Inconsistencies in the Data

The third discussion point concerns inconsistencies in the data. As a follow up to the discussion in the previous section, a cause for concern is as follows: looking at Table 11, entry #18 and entry #19, corresponding to *t_fox,m1(pmd/sub)_* and *t_fox,m2(pmd/sub)_*, respectively, should be equal, according to the equalities presented in Fig. 8, due to similar processing before the m1 deposition. The *u_c_* values are such that for #18 the electrical approach is preferred and for #19 the physical approach is preferred. The preferred thicknesses differ by 0.102 μm when they should be equal. Even more significant, the electrical thicknesses are inconsistent with one another.

In addition to the above example, it is interesting that the glass layer (i.e., #13 in Table 11) does not have a very high *u_c_* value in comparison to the other thicknesses. This may be due to it being considered a local measurement and having only one uncertainty component, due to the uncertainty of the measurement of *step3_AB_(4)*^−^ in the calculation of *t_(gl)phys_* in Eq. (63), in the *u_c_* calculation.

### 7.4 Is a More Detailed Error Analysis Required?

The question arises, is a more detailed error analysis required? To begin addressing this question, look at Tables 11 and 12. These tables are comprehensive rank-orderings of the *u_c_* values from smallest to largest. A conventional way to determine if a more detailed analysis is required, is to compare the difference between the physical result and the electrical result (namely, *t_phys_* − *t_elec_*) with the uncertainty of the difference (namely, *u_diff_*) where

$$u_{diff}=\sqrt{u_{c,phys}^{2}+u_{c,elec}^{2}}.$$ (65)

This is done in the last column of Tables 11 and 12 using the *E_n_* statistic [15,16], which is calculated using the following equation:

$$E_{n}=\frac{\left|t_{phys}-t_{elec}\right|}{2u_{diff}}.$$ (66)

Of the 42 thicknesses in Tables 11 and 12, there are 11 thicknesses with *E_n_* values less than or equal to 1.0. On the flip side, there are 18 thicknesses with *E_n_* values greater than 1.0. The remaining thicknesses do not address this question because the thicknesses were assumed to be equal or only one thickness is presented. If the uncertainties of the measurements in Tables 11 and 12 were fully characterized and the results being compared were independent, we might expect about 95 % (perhaps 27 or 28 of the 29 results) to have an *E_n_* value less than or equal to 1.0. However, since there are only 11 thicknesses with *E_n_* values less than or equal to 1.0, it is likely that the uncertainties of these measurements have not been fully characterized and, in particular, that the assumptions we have made in Secs. 4.3, 5.1, 5.10, and Sec. 6 require further investigation.

### 7.5 Adding Uncertainty Components

Should more uncertainty components be added to the physical approach, the electrical approach, or both? For the physical approach, more uncertainty components can be added to address one or more of the many assumptions made in Secs. 4.3, 5.1, 5.10, and Sec. 6. For the electrical approach, more uncertainty components can be added to account for the assumptions in Secs. 4.3 and 5.1, modeling, edge effects, thickness inhomogeneities, calibration, repeatability, drift, and for noise in the capacitance measurement.

For the physical approach, we will start in Sec. 7.5.1 by adding an additional uncertainty component, *u_Lstep_*, to the step height measurements. This uncertainty component is due to the measurement uncertainty across the length of the two 50 μm long platforms involved in the step. Then, for the electrical approach, we will add in Sec. 7.5.2 an additional uncertainty component (which is larger for the larger thicknesses) to the thicknesses obtained from capacitance measurements. And lastly, in Sec. 7.5.3, the results will be assimilated to determine the preferred thicknesses for use in calculations.

As an aside, it should be mentioned that a thorough thickness analysis would add to the nomenclature a distinction between thicknesses obtained over field oxide and thicknesses obtained over active area. As such, #9 in Table 11 (as an example) would be split into two rows since the m1-to-p2 thickness was measured over field oxide for the electrical approach (as indicated in the first note beneath Table 6) and measured over active area for the physical approach (as determined from *step2_BD_*, shown in Fig. 2b, which is over active area). They should not be expected to be equal, which is counter to the second assumption in Sec. 4.3. Due to this, uncertainties can be added to assumptions, such as the ones given in Eqs. (21) and (22).

Along the same lines, since the electrical approach deals mainly with thicknesses over field oxide, TS #2 and TS #3 could also be designed over field oxide (thereby creating TS #4 and TS #5) to more appropriately compare similar thicknesses without relying on an assumption.

#### 7.5.1 Adding an Uncertainty Component to the Physical Approach

For the physical thicknesses, Table 15 for step height measurements and their uncertainties was constructed. It is an extension of Table 5 in that an additional uncertainty component (namely *u_Lstep_*) is included in the calculation of *u_c_* for step heights. The uncertainty component, *u_Lstep_*, is due to the measurement uncertainty across the length of the two 50 μm long platforms involved in the step. Consult Sec. A.4 for more details concerning the other column headings in this table.

Examining Table 15, the uncertainty components *u_Wstep_* (the measurement uncertainty across the 100 μm width of the step) and *u_Lstep_* can be associated with non-uniformity issues across one or both of the platforms involved in the step. Notice that the values for *u_Lstep_* are comparable to the values for *uWstep* and recall that *u_Lstep_* was not included in the uncertainty analysis that was done earlier in this paper. Had we included *u_Lstep_* into the uncertainty analysis that was done earlier, which only included *u_Wstep_* and *u_basic_*, as explained in Sec. A.4, the results for which are presented in Tables 11 and 12, there would have been little overall effect in that the same thicknesses would be preferred as specified by the highlighted values in these tables. In addition, there would still be 11 thicknesses with *E_n_* values less than or equal to 1.0 and 18 thicknesses with *E_n_* values greater than 1.0.

#### 7.5.2 Adding an Uncertainty Component to the Electrical Approach

Recall from Sec. 7.2 that the electrical measurements in #4 and #5 of Table 10 differ by approximately 0.249 μm when they should be equal. We will use this as a starting point for obtaining uncertainties for thicknesses obtained from capacitances. The largest thickness in Table 6 (that is, #10) is 2.300 μm for [*t_fox,m2(pmdsub)_* + *t_pmd(imd/fox)_* + *t_imd(m2/pmd)_*]*_elec_*. Ten percent (or 0.230 μm) of this thickness is comparable to the 0.249 μm difference obtained. Since an uncertainty of the thickness (*u_res_*) divided by the thickness (*t*) is equal to a standard deviation of the capacitance (*σ_resCa_*) divided by the capacitance (*C_a_*), or rearranged

$$u_{res}=\frac{\sigma_{resCa}t}{C_{a}},$$ (67)

then with *t* = 2.300 μm, *u_res_* = 0.230 μm, and *C_a_* = 15.0 aF/μm^2^, it follows that *σ_resCa_* = 1.5 aF/μm^2^. This value for *σ_resCa_* can be used with all the capacitances to obtain the additional residual uncertainty component, *u_res_*, for the other thicknesses using Eq. (67). The results of including *u_res_* as an uncertainty component are given in Table 16. Note that *u_res_* gets larger as the capacitance gets smaller and that it is negligible for the largest capacitance.

#### 7.5.3 The Preferred Thicknesses

Given the above analysis, Tables 17 and 18 are the revised rank-orderings of the *u_c_* values for all the thicknesses, comparable to Tables 11 and 12. For the entries in Tables 11 and 17, the inclusion of the above uncertainties for the physical approach and the electrical approach, presented in Secs. 7.5.1 and 7.5.2, had the effect of switching 2 thicknesses {namely, *t_fox,m1(pmd/sub)_* and [*t_fox,m1(pmd/sub)_* + *t_pmd(m1/fox)_* from being a preferred electrical thickness to being a preferred physical thickness and switching 1 thickness [namely, *t_(m1)_*] from being a preferred physical thickness to being a preferred electrical thickness. The *u_c_* value below which electrical measurements are typically preferred has shifted from being less than or equal to 0.011 μm as ascertained in Table 11 to being less than or equal to 0.035 μm as ascertained in Table 17, for those thicknesses for which an electrical thickness can be obtained. There does not appear to be a preferred approach for thicknesses with larger values of *u_c_*, it depends upon the particular thickness. Now, 23 of the thicknesses have *E_n_* values less than or equal to 1.0, and 6 *E_n_* values greater than 1.0. Plus, as pertains to Sec. 7.3, the two preferred values for the thicknesses *t_fox,m1(pmd/sub)_* and *t_fox,m2(pmd/sub)_* as given in #15 and #16 of Table 17, which should be equal, differ by only 0.012 μm and their *E_n_* values are less than or equal to 1.0 (as opposed to the previous difference of 0.102 μm with *E_n_* values greater than 1.0).

Considering there are 23 (and not 27 or 28) thicknesses out of 29 thicknesses with *E_n_* values less than or equal to 1.0, there still seem to be sources of uncertainty that are unaccounted for. This suggests that further uncertainty analysis is required and that the effects of several assumptions need to be quantified. Further analysis of the correlations between the calculated thickness results should also be performed.

## 8. Conclusion

In conclusion, the electro-physical technique was presented which combines a physical approach and an electrical approach for determining the layer thicknesses for a 1.5 μm commercial CMOS process. The physical approach obtains thicknesses from step-height measurements on thickness test structures. The designs for these test structures were given in Figs. 1, 2, and 3. The electrical approach obtains thicknesses from capacitances, sheet resistances and resistivities, crystal lattice calculations, and the equating of similar oxides between platforms. The thickness from the approach that results in the lower value for *u_c_* is the reported value, meaning this number is chosen, for example, for use in Young’s modulus calculations.

Due to the apparent inconsistencies between the physical approach and the electrical approach for 18 thicknesses (as determined by the number of *E_n_* values greater than 1.0 in Tables 11 and 12), an additional uncertainty component was added to the physical step height measurements and to the electrical capacitance measurements in order to produce consistent results. This resulted in 23 (and not 27 or 28) thicknesses out of 29 thicknesses with *E_n_* values less than or equal to 1.0, which is an improvement over 11 *E_n_* values being less than or equal to 1.0. However, this still suggests that further uncertainty analysis is required and that the effects of several assumptions need to be quantified. For the processing run under consideration, the reported values are highlighted in Tables 17 and 18.

As a rule of thumb, given the results in Table 17, the electrical approach is preferred for thicknesses with *u_c_* values less than or equal to 0.035 μm. This corresponds somewhat to the layers, such as the poly2-to-poly1 oxide and the poly2 layers, which tend to be fabricated earlier in the processing sequence. The capacitances for the oxide layers fabricated earlier in the processing sequence are typically higher and thus easier to measure accurately. For capacitances less than or equal to 24.7 aF/μm^2^, the physical approach is preferred, as seen in Table 16.

The electro-physical technique enabled us to calculate the thicknesses of all the layers in the 1.5 μm commercial CMOS foundry process. Although some of the uncertainties appear to be small, the approach has provided thickness values that have been supported [6] via the successful optimization of the Young’s modulus values for the various layers in the process.

In the future as equipment and processes improve, an opto-electro-physical technique using spectroscopic ellipsometry in conjunction with this electro-physical technique has the potential to provide better results. Meanwhile, further analyses are required of the assumptions and uncertainties of these techniques.

## 9. Appendix A. Measurement Specifics and Uncertainty Calculations

In this appendix, measurement specifics and the equations used to determine the values of the combined standard uncertainty, *u_c_*, are presented. The first section presents the basic combined standard uncertainty equation, the second section presents platform-height measurement specifics and the more specific uncertainty equations for these measurements, the third section presents step-height measurement specifics and the corresponding uncertainty equations, the fourth section presents an additional uncertainty component for step-height measurements, and the fifth section presents the uncertainties for the thicknesses derived from capacitance and sheet resistance values.

### 9.1 The Combined Standard Uncertainty Equation

The combined standard uncertainty is comparable to the estimated standard deviation of the result [17]. It is equal to the square root of the sum of the squares of the uncertainty components. As pertains to this paper, the combined standard uncertainty can be calculated for thicknesses, platform-height measurements, and step-height measurements.

If there are three sources of uncertainty, there would be three uncertainty components, and the uncertainty equation would be as follows:

$$u_{c}=\sqrt{u_{1}^{2}+u_{2}^{2}+u_{3}^{2}}$$ (68)

where *u_1_* is the uncertainty component due to one of the sources of uncertainty, *u_2_* is the uncertainty component due to the second source of uncertainty, and *u_3_* is due to the third source of uncertainty. Additional components are added to Eq. (68) for each additional source of uncertainty, and similarly, if there are only two sources of uncertainty, the third term in Eq. (68) is removed.

As an example, consider the thickness formula in Eq. (32) involving two thicknesses as given below:

$$t_{fox(p2/sub)phys}=t_{fox,ab(p2/sub)phys}+t_{fox,be(p2/sub)phys}.$$ (69)

In Tables 9 and 10, the combined standard uncertainty values for *t_fox,ab(p2/sub)phys_* and *t_fox,be(p2/sub)phys_* are 0.0053 μm and 0.0103 μm, respectively. Therefore, we can calculate *u_c_* for *t_fox(p2/sub)phys_* as follows:

$$u_{c}=\sqrt{{\left(0.0053\right)}^{2}+{\left(0.0103\right)}^{2}}=0.0116\;\mu m.$$ (70)

This value for *u_c_* is presented in #2 of Table 8. Many of the other uncertainties are calculated in a similar manner.

Uncertainty components undergo either a Type A or Type B evaluation [17]. A Type A evaluation is based on any valid statistical method. A Type B evaluation includes experience with the behavior of relevant materials and instruments, for example. Therefore, the type of distribution (for example, a Gaussian or uniform distribution) is typically specified for Type B evaluations. For all of the uncertainty components in this paper, a Type B evaluation is assumed with a Gaussian distribution unless otherwise specified.

### 9.2 Measurement Specifics and Uncertainties for Platform-Height Measurements

In this section, the measurement specifics are presented for the platform-height measurements in Table 2 along with the combined standard uncertainty equation associated with this measurement.

For the platform-height measurements in Table 2, interferometric 3-D data sets are obtained incorporating the pertinent platforms on the thickness test structures.^13^ The data sets are leveled with respect to the reference platform. Therefore, the height of the reference platform is at or near zero.

**Fig. 19:**
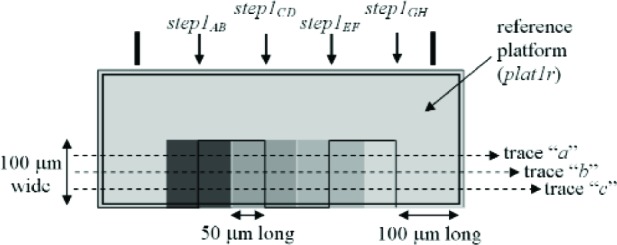
A design rendition of thickness TS #1 indicating the location of the 2-D data traces obtained for interferometric measurements.

**Fig. 20:**
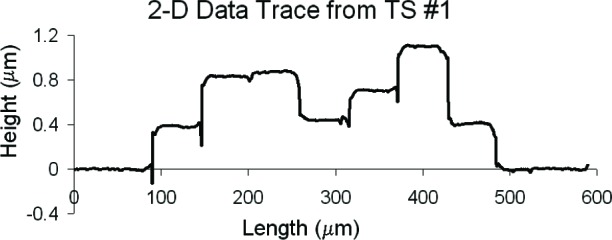
A 2-D data trace extracted from TS #1.

Three 2-D data traces (such as, traces “*a*,” “*b*,” and “*c*” shown in Fig. 19) are extracted from an interferometric 3-D data set and calibrated. These data traces (such as the one shown in Fig. 20) traverse the platforms, each being 50 μm long and 100 μm wide (except for the reference platforms which are 100 μm long). Therefore, for each 2-D data trace, data from approximately 70 μm in the center of each 100 μm long reference platform is averaged and data from approximately 20 μm in the center of each 50 μm long platform is averaged.

The reference platform height [12], *platNr*, is the average of the values obtained from the reference platforms from all the data traces. [The standard deviation (*splatNr*) of these values is also calculated.] A calculation for *platNr* is given below:

$$platNrW=\frac{platNrWa+platNrWb+platNrWc}{3}$$ (71)

$$platNrE=\frac{platNrEa+platNrEb+platNrEc}{3}$$ (72)

$$platNr=\frac{platNrW+platNrE}{2}$$ (73)

where an “*a*,” “*b*,” or “*c*” appendage to the platform designator refers to the data trace (“*a*,” “*b*,” “*c*,” etc.) the measurement was taken from.

For each 50 μm long platform, the three measurements taken from the platform from traces “*a*,” “*b*,” and “*c*” are averaged together (and the standard deviation, *s_platNX_*, is calculated) and the reference platform height, *platNr*, is subtracted from this average to obtain the platform-height measurement [12], *platNX*, as given below:

$$platNX=\frac{platNXa+platNXb+platNXc}{3}-platNr.$$ (74)

The measurement uncertainty across the width of the platform, *u_WplatNX_*, is equal to the square root of the sum of the squares of *s_platNX_* and *s_platNr_*, namely:

$$u_{WplatNX}=\sqrt{s_{platNX}{}^{2}+s_{platNr}{}^{2}}.$$ (75)

This is the first component in the combined standard uncertainty equation given below for platform-height measurements:

$$u_{platNX}=\sqrt{u_{WplatNX}{}^{2}+u_{cert}{}^{2}+u_{repeat}{}^{2}+u_{drift}{}^{2}+u_{linear}{}^{2}}$$ (76)

where *u_cert_* is the component in the combined standard uncertainty calculation that is due to the uncertainty of the value of the step-height standard, *u_repeat_* is the uncertainty of instrument calibration due to the repeatability of the measurements of the calibration standard, *u_drift_* is the uncertainty of a measurement due to the amount of drift during the data session^14^, and *u_linear_* is the uncertainty of a measurement due to the deviation from linearity of the data scan. (See [18] for the equations associated with these uncertainty components.) In particular, we can write the following equation for *u_cert_* assuming a Gaussian distribution:

$$u_{cert}=\frac{\sigma_{cert}}{cert}\left|platNX\right|$$ (77)

where *platNX* is the calibrated platform-height measurement under consideration, *cert* is the certified value of the step-height standard, and *σ_cert_* is the certified one sigma uncertainty of the certified step-height standard. For the presented data set, *cert* equals 9.887 μm and *σ_cert_* equals 0.083 μm.

We can write the following equation for *u_repeat_* assuming a uniform distribution:

$$u_{repeat}=\frac{z_{repeat}}{2(1.732){\bar{z}}_{6}}\left|platNX\right|$$ (78)

where *z_repeat_* is calculated to be the maximum of two uncalibrated values; one of which is the positive difference between the minimum and maximum values of the six calibration measurements taken before the data session (at the same location on the step-height standard) and the other is the positive difference between the minimum and maximum values of the six calibration measurements taken after the data session (at the same location on the step-height standard). Also, ${\bar{z}}_{6}$ is the uncalibrated average of the six calibration measurements from which *z_repeat_* is found. For the presented data set, *z_repeat_* equals 0.029 μm and ${\bar{z}}_{6}$ equals 9.821 μm. However, for the calculations involving *plat3D(0)*, *z_repeat_* equals 0.035 μm and ${\bar{z}}_{6}$ equals 9.774 μm, since the measurement of *plat3D(0)* was taken during a different data session.

We can make the following calculation for *u_drift_* assuming a uniform distribution:

$$u_{drift}=\frac{z_{drift}(cal_{z})}{2(1.732)cert}\left|platNX\right|$$ (79)

where *z_drift_* is calculated as follows: the average of the six calibration measurements taken before the data session (at the same location on the step-height standard) is determined and the average of the six calibration measurements taken after the data session (at the same location on the step-height standard) is determined. Then, *z_drift_* is calculated as the positive uncalibrated difference between these two values. Also, *cal_z_* is the *z*-calibration factor which is equal to *cert* divided by the average of the twelve calibration measurements. For the presented data set, *z_drift_* equals 0.013 μm and *cal_z_* equals 1.0061. However, for the calculations involving *plat3D(0)*, *z_drift_* equals 0.010 μm and *cal_z_* equals 1.0121.

And lastly, we can make the following calculation for *u_linear_* assuming a uniform distribution:

$$u_{linear}=\frac{z_{perc}}{1.732}\left|platNX\right|$$ (80)

where *z_perc_* is the percent quoted by the interferometer manufacturer as the maximum deviation from linearity over the *z*-scan range. For the presented data set, *z_perc_* equals 1.0 %.

The resulting calculated values of *u_c_* are presented in Table 2.

### 9.3 Measurement Specifics and Uncertainties for Step-Height Measurements

In this section, the measurement specifics will be presented for step-height measurements along with the combined standard uncertainty equations associated with this measurement.

For step-height measurements [12] taken from one 3-D data set, given the averaged data from approximately 20 μm in the center of each 50 μm long platform for each data trace (as detailed in the previous section), the following calculation is used for each data trace:

$$stepN_{XYt}=platNYt-platNXt$$ (81)

where *t* is the data trace (“*a*,” “*b*,” “*c*,” etc.) being examined. The step-height measurement is then calculated to be the average of these measurements as follows:

$$stepN_{XY}=\frac{stepN_{XYa}+stepN_{XYb}+stepN_{XYc}}{3}.$$ (82)

The standard deviation of these step-height measurements, *s_stepNXY_*, is also calculated and the following equality can be made:

$$u_{Wstep}=s_{stepNXY}$$ (83)

where *u_Wstep_* is the first component in the combined standard uncertainty equation for step-height measurements and it is due to the measurement uncertainty of the step height across the width of the step. This combined standard uncertainty equation, a modification of Eq. (76), is given below for the step-height measurements presented in Table 5:

$$u_{c}=\sqrt{u_{Wstep}{}^{2}+u_{cert}{}^{2}+u_{repeat}{}^{2}+u_{drift}{}^{2}+u_{linear}{}^{2}}$$ (84)

where *platNX* gets replaced with *stepN_XY_* in the calculations of *u_cert_*, *u_repeat_*, *u_drift_*, and *u_linear_* in Eqs. (77) to (80), respectively.

If two different 3-D data sets (one data set including the first platform, *platNX*, and a reference platform and the other data set including the second platform, *platNY* or *platMY*, and a reference platform) are used to find *stepN_XY_* or *stepN_X_M_Y_* (where *N* is the test structure number associated with *platNX* and *M* is the test structure number associated with *platMY*) then *stepN_XY_* or *stepN_X_M_Y_* is found using one of the following equations:

$$stepN_{XY}=platNY-platNX$$ (85)

or

$$stepN_{X}M_{Y}=platMY-platNX$$ (86)

where *platNX*, *platNY*, and *platMY* are found using Eq. (74).

With two different 3-D data sets, Eq. (84) is not used to find the combined standard uncertainty for step-height measurements. Instead, the combined standard uncertainty equation for *stepN_XY_* or *stepN_X_M_Y_* would be as follows:

$$u_{c}=\sqrt{u_{platNX}{}^{2}+u_{platNY}{}^{2}}$$ (87)

or

$$u_{c}=\sqrt{u_{platNX}{}^{2}+u_{platMY}{}^{2}}$$ (88)

where *u_platNX_* is the uncertainty component due to the measurement of *platNX*, *u_platNY_* is the uncertainty component due to the measurement of *platNY*, and *u_platMY_* is the uncertainty component due to the measurement of *platMY*, each component of which was found using Eq. (76).

In summary, using Eqs. (87) or (88) would typically produce a larger value for *u_c_*, than if Eq. (84) were used. Therefore, whenever possible, step-height measurements are taken and Eq. (84) used as opposed to the use of platform-height measurements to determine step heights.

### 9.4 An Additional Uncertainty Component for Step-Height Measurements

An additional uncertainty component (namely *uLstep*) can be added to Eq. (84) in the calculation of *u_c_* for step-height measurements producing the following equation:

$$u_{c}=\sqrt{u_{basic}{}^{2}+u_{Wstep}{}^{2}+u_{Lstep}{}^{2}}$$ (89)

where the components *u_basic_*, *u_Wstep_*, and *u_Lstep_* are described in the next few paragraphs. Table 15, an extension of Table 5 for step-height measurements, includes these uncertainty components.

The first component, *u_basic_*, in Eq. (89), presented in column 6 of Table 15, includes all the basic interferometric-related uncertainty components. In other words,

$$u_{basic}=\sqrt{u_{cert}{}^{2}+u_{repeat}{}^{2}+u_{drift}{}^{2}+u_{linear}{}^{2}}$$ (90)

for which the components *u_cert_*, *u_repeat_*, *u_drift_*, and *u_linear_* are also found in Table 5. Consult Sec. A.3 for details associated with these components.

The second component, *u_Wstep_*, in Eq. (89) presented in column 7 of Table 15, is due to the measurement uncertainty of the step-height across the 100 μm width of the step. Column 7 in Table 15 is a replica of column 6 in Table 5. Therefore, the two components *u_basic_* and *u_Wstep_* produce the values of *u_c_* given in Table 5.

The third component, *u_Lstep_*, in Eq. (89) presented in column 8 of Table 15, is the new uncertainty component. It is due to the measurement uncertainty of the step height across the length of the two 50 μm long platforms involved in the step. It is calculated using the following formula:

$$u_{LstepNXY}=\sqrt{{\left(\sigma_{platNX}-\sigma_{rough}\right)}^{2}+{\left(\sigma_{platNY}+\sigma_{rough}\right)}^{2}}$$ (91)

where *σ_platNX_*, also found in column 4 of Table 15, is extracted from a 2-D data trace, such as trace “*b*” in Fig. 19, ideally taken through both platforms involved in the step. It is the standard deviation of the interferometric measurements taken from approximately 20 μm along the data trace in the center of the 50 μm long platform, *platNX*. Similarly, *σ_platNY_*, also found in column 5, is taken from approximately 20 μm along the data trace in the center of the 50 μm long platform, *platNY*. Note in Eq. (91) that the value for *σ_rough_* gets subtracted from *σ_platNX_* and *σ_platNY_*, where *σ_rough_* is the smallest of all the values for *σ_platNX_* and *σ_platNY_* obtained in this analysis. For the presented data set, *σ_rough_* equals 0.0025 μm.

### 9.5 Uncertainties for the Electrical Approach

In this section, the equations will be presented for the combined standard uncertainty values presented in Table 6 for the thicknesses derived from the MOSIS-supplied capacitance values, and in Table 7 for the thicknesses derived from the MOSIS-supplied sheet resistance values.

For the combined standard uncertainty calculations of the thicknesses derived from the MOSIS-supplied capacitance values, look at Eq. (2). There are two uncertainty components; one due to the uncertainty of *C_a_*, or *u_Ca_*, and one due to the uncertainty of *ε_SiO2_*, or *u_ε_*, such that

$$u_{c}=\sqrt{u_{Ca}{}^{2}+u_{\varepsilon }{}^{2}}.$$ (92)

The first uncertainty component, *u_Ca_*, is assumed to be due solely to the standard deviation of the MOSIS-supplied capacitance values, *σ_Ca_*.^15^ The capacitance values (*C_a_*) and their standard deviations (*σ_Ca_*) as supplied by MOSIS are presented in Table 6. Basically, with the help of Eq. (2)*u_Ca_* is calculated as follows assuming a Gaussian distribution:

$$t\left(C_{a}\right)=\frac{\varepsilon_{SiO2}}{C_{a}}$$ (93)

$$t\left(C_{a}+\sigma_{Ca}\right)=\frac{\varepsilon_{SiO2}}{C_{a}+\sigma_{Ca}}$$ (94)

$$t\left(C_{a}-\sigma_{Ca}\right)=\frac{\varepsilon_{SiO2}}{C_{a}-\sigma_{Ca}}$$ (95)

$$u_{Ca}=\frac{\left|t\left(C_{a}+\sigma_{Ca}\right)-t\left(C_{a}-\sigma_{Ca}\right)\right|}{2}.$$ (96)

The second uncertainty component, *u_ε_* in Eq. (92), is calculated given *σ_e_*, which is assumed to be 0.1 aF/μm (which is the uncertainty of the last digit of the constant *ε_SiO2_* which equals 34.5 aF/μm). Therefore, with the help of Eq. (2)*u_ε_* is calculated as follows assuming a uniform distribution:

$$t\left(\varepsilon_{SiO2}+3\sigma_{\varepsilon }\right)=\frac{\varepsilon_{SiO2}+3\sigma_{\varepsilon }}{C_{a}}$$ (97)

$$t\left(\varepsilon_{SiO2}-3\sigma_{\varepsilon }\right)=\frac{\varepsilon_{SiO2}-3\sigma_{\varepsilon }}{C_{a}}$$ (98)

$$u_{\varepsilon }=\frac{\left|t\left(\varepsilon_{SiO2}+3\sigma_{\varepsilon }\right)-t\left(\varepsilon_{SiO2}-3\sigma_{\varepsilon }\right)\right|}{2(1.732)}.$$ (99)

For the combined standard uncertainty calculations of the thicknesses derived from the MOSIS-supplied sheet resistance values, look at Eq. (3). There are two uncertainty components; one due to the uncertainty of *R_s_*, or *u_Rs_*, and one due to the uncertainty of *ρ*, or *u_ρ_*, such that

$$u_{c}=\sqrt{u_{Rs}{}^{2}+u_{\rho }{}^{2}}.$$ (100)

The first uncertainty component, *u_Rs_*, is assumed to be due solely to the standard deviation of the MOSIS-supplied sheet resistance values, *σ_Rs_*. The sheet resistances (*R_s_*) and their standard deviations (*σ_Rs_*) are presented in Table 7. With the help of Eq. (3)*u_Rs_* is calculated as follows assuming a Gaussian distribution:

$$t\left(R_{s}\right)=\frac{\rho }{R_{s}}$$ (101)

$$t\left(R_{s}+\sigma_{Rs}\right)=\frac{\rho }{R_{s}+\sigma_{Rs}}$$ (102)

$$t\left(R_{s}-\sigma_{Rs}\right)=\frac{\rho }{R_{s}-\sigma_{Rs}}$$ (103)

$$u_{Rs}=\frac{\left|t\left(R_{s}+\sigma_{Rs}\right)-t\left(R_{s}-\sigma_{Rs}\right)\right|}{2}.$$ (104)

The second uncertainty component, *u_ρ_* in Eq. (100), is calculated given *σ_ρ_*, which is assumed to be 0.1 Ω μm for p1 and p2 and 0.001 Ω μm for m1 and m2 (which is the uncertainty of the last digit of the resistivities). Therefore, with the help of Eq. (3)*u_ρ_* is calculated as follows assuming a uniform distribution:

$$t\left(\rho +3\sigma_{\rho }\right)=\frac{\rho +3\sigma_{\rho }}{R_{s}}$$ (105)

$$t\left(\rho -3\sigma_{\rho }\right)=\frac{\rho -3\sigma_{\rho }}{R_{s}}$$ (106)

$$u_{\rho }=\frac{\left|t\left(\rho +3\sigma_{\rho }\right)-t\left(\rho -3\sigma_{\rho }\right)\right|}{2(1.732)}.$$ (107)

## Figures and Tables

**Table 1:** Nomenclature for the interconnect and oxide layers

#	Thickness designation	Interconnect or oxide layer	Top layer	Bottom layer
1	*t_(p1)_*	poly1 (p1)	–	–
2^1^	*t_(p1′)_*	reduced poly1 (p1′)	–	–
3	*t_(p2)_*	poly2 (p2)	–	–
4	*t_(m1)_*	metal1 (m1)	–	–
5	*t_(m2)_*	metal2 (m2)	–	–
6	*t_(gl)_*	glass (gl)	–	–
7	*t_(ni)_*	nitride cap (ni)	–	–

8	*t_fox(p1/sub)_*	fox	p1	sub
9	*t_fox(p2/sub)_*	fox	p2	sub
10	*t_fox(pmd/sub)_*	fox	pmd	sub
11	*t_thin(p1/aan)_*	thin	p1	aan
12	*t_thin(p2/aan)_*	thin	p2	aan
13	*t_thin(p2/p1)_*	thin	p2	p1
14	*t_pmd(m1/aan)_*	pmd	m1	aan
15	*t_pmd(m1/fox)_*	pmd	m1	fox
16	*t_pmd(m1/p1)_*	pmd	m1	p1
17	*t_pmd(m1/p2)_*	pmd	m1	p2
18	*t_pmd(imd/aan)_*	pmd	imd	aan
19	*t_pmd(imd/fox)_*	pmd	imd	fox
20	*t_pmd(imd/p1)_*	pmd	imd	p1
21	*t_pmd(imd/p2)_*	pmd	imd	p2
22	*t_imd(m2/m1)_*	imd	m2	m1
23	*t_imd(m2/pmd)_*	imd	m2	pmd
24	*t_imd(gl/m1)_*	imd	gl	m1
25	*t_imd(gl/pmd)_*	imd	gl	pmd

1 This thickness refers to the first polysilicon layer (poly1) thickness if a thermal oxide is grown on top of it.

**Table 2:** Platform-height measurements

#	Platform^1^	From^2^	To^2^	Height^3^ (μm)	*s_platNX_* (μm)	*s_platNr_* (μm)	*u_cert_* (μm)	*u_repeat_* (μm)	*u_drift_* (μm)	*u_linear_* (μm)	*u_c_* (μm)
1	*plat1rW*	41	41	−0.0020	0.0017	–	0.0000	0.00000	0.00000	0.0000	0.0017
2	*plat1A*	41	42	0.3860	0.0025	0.0027	0.0032	0.00033	0.00015	0.0022	0.0054
3	*plat1B*	41	18	0.840	0.0067	0.0027	0.0071	0.00072	0.00032	0.0048	0.011
4	*plat1C*	41	19	0.8763	0.0020	0.0027	0.0074	0.00075	0.00033	0.0051	0.0096
5	*plat1D*	41	43	0.4467	0.0010	0.0027	0.0037	0.00038	0.00017	0.0026	0.0054
6	*plat1E*	41	45	0.710	0.0066	0.0027	0.0060	0.00061	0.00027	0.0041	0.010
7	*plat1F*	41	21	1.110	0.0096	0.0027	0.0093	0.00095	0.00042	0.0064	0.015
8	*plat1G*	41	17	0.4128	0.0025	0.0027	0.0035	0.00035	0.00016	0.0024	0.0056
9	*plat1rE*	41	41	0.0000	0.0035	–	0.0000	0.00000	0.00000	0.0000	0.0035
10	*plat2A*	41	45	0.7296	0.0023	0.0021	0.0061	0.00062	0.00028	0.0042	0.0081
11	*plat2B*	41	46	1.128	0.0091	0.0021	0.0095	0.00096	0.00043	0.0065	0.015
12	*plat2C*	41	48	1.608	0.0136	0.0021	0.0135	0.00137	0.00061	0.0093	0.021
13	*plat2D*	41	47	1.189	0.0071	0.0021	0.0100	0.00101	0.00045	0.0069	0.014
14	*plat2E*	41	43	0.4546	0.0030	0.0048	0.0038	0.00039	0.00017	0.0026	0.0073
15	*plat3A(0)*	41	41	0.0000	0.0021	–	0.0000	0.00000	0.00000	0.0000	0.0021
16	*plat3A(4)*	41	41	0.0000	0.0057	–	0.0000	0.00000	0.00000	0.0000	0.0057
17	*plat3B(0)*	41	33	1.180	0.0096	0.0021	0.0099	0.00101	0.00045	0.0068	0.016
18	*plat3B(4)*	41	33	0.4876	0.0025	0.0057	0.0041	0.00042	0.00019	0.0028	0.0080
19	*plat3C(0)*	41	29	0.7609	0.0044	0.0021	0.0064	0.00065	0.00029	0.0044	0.0092
20	*plat3D(0)*^4^	41	37	1.933	0.0126	0.0021	0.0162	0.00200	0.00057	0.0112	0.024

1 These platform-height measurements are not required in the analysis. However, they are included if two 3-D data sets are used to obtain a step-height measurement and if the reader would like to perform additional calculations.

2 The entries in these columns refer to the design numbers in Tables 3 and 4.

3 The measurements on TS #1 and TS #2 were taken on an unetched chip. The measurements on TS #3 were taken on chips covered with chromium and gold on either an unetched chip (as indicated by a “*0*” in parenthesis following the platform name) or on a chip post-processed using 4 cycles of a XeF_2_ etch (as indicated by a “*4*” in parenthesis) which removed the nitride cap.

4 This measurement was taken during a different data session. Also, it was a measurement that was taken over field oxide and adjusted for use over active area.

**Table 3:** Thickness layers over field oxide

#	fox	p1	thin	p2	pmd	m1	imd	m2	gl	ni^1^
1	*t_fox(pmd/sub)_*	–	–	–	*t_pmd(imd/fox)_*	–	*t_imd(gl/pmd)_*	–	*t_(gl)_*	*t_(ni)_*
2	*t_fox(p1/sub)_*	*t_(p1)_*	–	–	*t_pmd(imd/p1)_*	–	*t_imd(gl/pmd)_*	–	*t_(gl)_*	*t_(ni)_*
3	*t_fox(p2/sub)_*	–	–	*t_(p2)_*	*t_pmd(imd/p2)_*	–	*t_imd(gl/pmd)_*	–	*t_(gl)_*	*t_(ni)_*
4	*t_fox(p1/sub)_*	*t_(p1_*_′_*_)_*	*t_thin(p2/p1)_*	*t_(p2)_*	*t_pmd(imd/p2)_*	–	*t_imd(gl/pmd)_*	–	*t_(gl)_*	*t_(ni)_*
5	*t_fox(pmd/sub)_*	–	–	–	*t_pmd(m1/fox)_*	*t_(m1)_*	*t_imd(gl/m1)_*	–	*t_(gl)_*	*t_(ni)_*
6	*t_fox(p1/sub)_*	*t_(p1)_*	–	–	*t_pmd(m1/p1)_*	*t_(m1)_*	*t_imd(gl/m1)_*	–	*t_(gl)_*	*t_(ni)_*
7	t*_fox(p2/sub)_*	–	–	*t_(p2)_*	*t_pmd(m1/p2)_*	*t_(m1)_*	*t_imd(gl/m1)_*	–	*t_(gl)_*	*t_(ni)_*
8	*t_fox(p1/sub)_*	*t_(p1_*_′_*_)_*	*t_thin(p2/p1)_*	*t_(p2)_*	*t_pmd(m1/p2)_*	*t_(m1)_*	*t_imd(gl/m1)_*	–	*t_(gl)_*	*t_(ni)_*
9	*t_fox(pmd/sub)_*	–	–	–	*t_pmd(imd/fox)_*	–	*t_imd(m2/pmd)_*	*t_(m2)_*	*t_(gl)_*	*t_(ni)_*
10	*t_fox(p1/sub)_*	*t_(p1)_*	–	–	*t_pmd(imd/p1)_*	–	*t_imd(m2/pmd_)*	*t_(m2)_*	*t_(gl)_*	*t_(ni)_*
11	*t_fox(p2/sub)_*	–	–	*t_(p2)_*	*t_pmd(imd/p2)_*	–	*t_imd(m2/pmd)_*	*t_(m2)_*	*t_(gl)_*	*t_(ni)_*
12	*t_fox(p1/sub)_*	*t_(p1_*_′_*_)_*	*t_thin(p2/p1)_*	*t_(p2)_*	*t_pmd(imd/p2)_*	–	*t_imd(m2/pmd)_*	*t_(m2)_*	*t_(gl)_*	*t_(ni)_*
13	*t_fox(pmd/sub)_*	–	–	–	*t_pmd(m1/fox)_*	*t_(m1)_*	*t_imd(m2/m1)_*	*t_(m2)_*	*t_(gl)_*	*t_(ni)_*
14	*t_fox(p1/sub)_*	*t_(p1)_*	–	–	*t_pmd(m1/p1)_*	*t_(m1)_*	*t_imd(m2/m1)_*	*t_(m2)_*	*t_(gl)_*	*t_(ni)_*
15	*t_fox(p2/sub)_*	–	–	*t_(p2)_*	*t_pmd(m1/p2)_*	*t_(m1)_*	*t_imd(m2/m1)_*	*t_(m2)_*	*t_(gl)_*	*t_(ni)_*
16	*t_fox(p1/sub)_*	*t_(p1_*_′_*_)_*	*t_thin(p2/p1)_*	*t_(p2)_*	*t_pmd(m1/p2)_*	*t_(m1)_*	*t_imd(m2/m1)_*	*t_(m2)_*	*t_(gl)_*	*t_(ni)_*
17	*t_fox(pmd/sub)_*	–	–	–	*t_pmd(imd/fox)_*	–	*t_imd(m2/pmd)_*	*t_(m2)_*	–	–
18	*t_fox(p1/sub)_*	*t_(p1)_*	–	–	*t_pmd(imd/p1)_*	–	*t_imd(m2/pmd)_*	*t_(m2)_*	–	–
19	*t_fox(p2/sub)_*	–	–	*t_(p2)_*	*t_pmd(imd/p2)_*	–	*t_imd(m2/pmd)_*	*t_(m2)_*	–	–
20	*t_fox(p1/sub)_*	*t_(p1_*_′_*_)_*	*t_thin(p2/p1)_*	*t_(p2)_*	*t_pmd(imd/p2)_*	–	*t_imd(m2/pmd)_*	*t_(m2)_*	–	–
21	*t_fox(pmd/sub)_*	–	–	–	*t_pmd(m1/fox)_*	*t_(m1)_*	*t_imd(m2/m1)_*	*t_(m2)_*	–	–
22	*t_fox(p1/sub)_*	*t_(p1)_*	–	–	*t_pmd(m1/p1)_*	*t_(m1)_*	*t_imd(m2/m1)_*	*t_(m2)_*	–	–
23	*t_fox(p2/sub)_*	–	–	*t_(p2)_*	*t_pmd(m1/p2)_*	*t_(m1)_*	*t_imd(m2/m1)_*	*t_(m2)_*	–	–
24	*t_fox(p1/sub)_*	*t_(p1_*_′_*_)_*	*t_thin(p2/p1)_*	*t_(p2)_*	*t_pmd(m1/p2)_*	*t_(m1)_*	*t_imd(m2/m1)_*	*t_(m2)_*	–	–

1 The nitride cap may or may not be present.

**Table 4:** Thickness layers over *n*-doped active area

#	thin	p1	thin	p2	pmd	m1	imd	m2	gl	ni^1^
25	–	–	–	–	*t_pmd(imd/aan)_*	–	*t_imd(gl/pmd)_*	–	*t_(gl)_*	*t_(ni)_*
26	*t_thin(p1/aan)_*	*t_(p1)_*	–	–	*t_pmd(imd/p1)_*	–	*t_imd(gl/pmd)_*	–	*t_(gl)_*	*t_(ni)_*
27	–	–	*t_thin(p2/aan)_*	*t_(p2)_*	*t_pmd(imd/p2)_*	–	*t_imd(gl/pmd)_*	–	*t_(gl)_*	*t_(ni)_*
28	*t_thin(p1/aan)_*	*t_(p1_*_′_*_)_*	*t_thin(p2/p1)_*	*t_(p2)_*	*t_pmd(imd/p2)_*	–	*t_imd(gl/pmd)_*	–	*t_(gl)_*	*t_(ni)_*
29	–	–	–	–	*t_pmd(m1/aan)_*	*t_(m1)_*	*t_imd(gl/m1)_*	–	*t_(gl)_*	*t_(ni)_*
30	*t_thin(p1/aan)_*	*t_(p1)_*	–	–	*t_pmd(m1/p1)_*	*t_(m1)_*	*t_imd(gl/m1)_*	–	*t_(gl)_*	*t_(ni)_*
31	–	–	*t_thin(p2/aan)_*	*t_(p2)_*	*t_pmd(m1/p2)_*	*t_(m1)_*	*t_imd(gl/m1)_*	–	*t_(gl)_*	*t_(ni)_*
32	*t_thin(p1/aan)_*	*t_(p1_*_′_*_)_*	*t_thin(p2/p1)_*	*t_(p2)_*	*t_pmd(m1/p2)_*	*t_(m1)_*	*t_imd(gl/m1)_*	–	*t_(gl)_*	*t_(ni)_*
33	–	–	–	–	*t_pmd(imd/aan)_*	–	*t_imd(m2/pmd)_*	*t_(m2)_*	*t_(gl)_*	*t_(ni)_*
34	*t_thin(p1/aan)_*	*t_(p1)_*	–	–	*t_pmd(imd/p1)_*	–	*t_imd(m2/pmd)_*	*t_(m2)_*	*t_(gl)_*	*t_(ni)_*
35	–	–	*t_thin(p2/aan)_*	*t_(p2)_*	*t_pmd(imd/p2)_*	–	*t_imd(m2/pmd)_*	*t_(m2)_*	*t_(gl)_*	*t_(ni)_*
36	*t_thin(p1/aan)_*	*t_(p1_*_′_*_)_*	*t_thin(p2/p1)_*	*t_(p2)_*	*t_pmd(imd/p2)_*	–	*t_imd(m2/pmd)_*	*t_(m2)_*	*t_(gl)_*	*t_(ni)_*
37	–	–	–	–	*t_pmd(m1/aan)_*	*t_(m1)_*	*t_imd(m2/m1)_*	*t_(m2)_*	*t_(gl)_*	*t_(ni)_*
38	*t_thin(p1/aan)_*	*t_(p1)_*	–	–	*t_pmd(m1/p1)_*	*t_(m1)_*	*t_imd(m2/m1)_*	*t_(m2)_*	*t_(gl)_*	*t_(ni)_*
39	–	–	*t_thin(p2/aan)_*	*t_(p2)_*	*t_pmd(m1/p2)_*	*t_(m1)_*	*t_imd(m2/m1)_*	*t_(m2)_*	*t_(gl)_*	*t_(ni)_*
40	*t_thin(p1/aan)_*	*t_(p1_*_′_*_)_*	*t_thin(p2/p1)_*	*t_(p2)_*	*t_pmd(m1/p2)_*	*t_(m1)_*	*t_imd(m2/m1)_*	*t_(m2)_*	*t_(gl)_*	*t_(ni)_*
41	–	–	–	–	*t_pmd(imd/aan)_*	–	*t_imd(m2/pmd)_*	*t_(m2)_*	–	–
42	*t_thin(p1/aan)_*	*t_(p1)_*	–	–	*t_pmd(imd/p1)_*	–	*t_imd(m2/pmd)_*	*t_(m2)_*	–	–
43	–	–	*t_thin(p2/aan)_*	*t_(p2)_*	*t_pmd(imd/p2)_*	–	*t_imd(m2/pmd)_*	*t_(m2)_*	–	–
44	*t_thin(p1/aan)_*	*t_(p1_*_′_*_)_*	*t_thin(p2/p1)_*	*t_(p2)_*	*t_pmd(imd/p2)_*	–	*t_imd(m2/pmd)_*	*t_(m2)_*	–	–
45	–	–	–	–	*t_pmd(m1/aan)_*	*t_(m1)_*	*t_imd(m2/m1)_*	*t_(m2)_*	–	–
46	*t_thin(p1/aan)_*	*t_(p1)_*	–	–	*t_pmd(m1/p1)_*	*t_(m1)_*	*t_imd(m2/m1)_*	*t_(m2)_*	–	–
47	–	–	*t_thin(p2/aan)_*	*t_(p2)_*	*t_pmd(m1/p2)_*	*t_(m1)_*	*t_imd(m2/m1)_*	*t_(m2)_*	–	–
48	*t_thin(p1/aan)_*	*t_(p1_*_′_*_)_*	*t_thin(p2/p1)_*	*t_(p2)_*	*t_pmd(m1/p2)_*	*t_(m1)_*	*t_imd(m2/m1)_*	*t_(m2)_*	–	–

1 The nitride cap may or may not be present.

**Table 5:** Step-height measurements

#	Step^1^	From	To	Height^2^ (μm)	*u_Wstep_* (μm)	*u_cert_* (μm)	*u_repeat_* (μm)	*u_drift_* (μm)	*u_linear_* (μm)	*u_c_* (μm)
1	*step1_AB_*	*plat1A*	*plat1B*	0.4538	0.0076	0.0038	0.00039	0.00017	0.0026	0.0089
2	*step1_CD_*	*plat1C*	*plat1D*	−0.4296	0.0027	0.0036	0.00037	0.00016	0.0025	0.0052
3	*step1_EF_*	*plat1E*	*plat1F*	0.4001	0.0031	0.0034	0.00034	0.00015	0.0023	0.0051
4	*step1_GH_*	*plat1G*	*plat1H*	−0.4118	0.0021	0.0035	0.00035	0.00016	0.0024	0.0047
5	*step1_rA_*	*plat1rW*	*plat1A*	0.3870	0.0032	0.0032	0.00033	0.00015	0.0022	0.0051
6	*step1_rD_*	*plat1rW*	*plat1D*	0.4477	0.0027	0.0038	0.00038	0.00017	0.0026	0.0053
7	*step1_rE_*	*plat1rW*	*plat1E*	0.7113	0.0050	0.0060	0.00061	0.00027	0.0041	0.0088
8	*step2_rA_*	*plat2rW*	*plat2A*	0.7304	0.0036	0.0061	0.00062	0.00028	0.0042	0.0083
9	*step2_AB_*	*plat2A*	*plat2B*	0.3988	0.0071	0.0033	0.00034	0.00015	0.0023	0.0082
10	*step2_BC_*	*plat2B*	*plat2C*	0.4796	0.0045	0.0040	0.00041	0.00018	0.0028	0.0067
11	*step2_CD_*	*plat2C*	*plat2D*	−0.4189	0.0065	0.0035	0.00036	0.00016	0.0024	0.0078
12	*step2_BD_*	*plat2B*	*plat2D*	0.0607	0.0021	0.00051	0.000052	0.000023	0.00035	0.0022
13	*step3_AB_(0)*	*plat3A(0)*	*plat3B(0)*	1.180	0.0081	0.0099	0.0010	0.00045	0.0068	0.015
14	*step3_AB_(4)*	*plat3A(4)*	*plat3B(4)*	0.4876	0.0071	0.0041	0.00042	0.00019	0.0028	0.0087
15	*step3_BC_(0)*	*plat3B(0)*	*plat3C(0)*	−0.419	0.0137	0.0035	0.00036	0.00016	0.0024	0.014

1 The step-height measurements in this table were obtained using one 3-D data set. If *step3_CD_(0)* were obtained using one 3-D data set, it also would be included and then this table would be comprised of all the step-height measurements required for a complete analysis.

2 The measurements on TS #1 and TS #2 were taken on an unetched chip. The measurements on TS #3 were taken on chips covered with chromium and gold on either an unetched chip (as indicated by a “*0*” in parenthesis following the platform and step name) or on a chip post-processed using 4 cycles of a XeF_2_ etch (as indicated by a “*4*” in parenthesis) which removed the nitride cap.

**Table 6:** Oxide thickness values from capacitances^1^,^2^

#	Top layer	Bottom layer	Thickness designation	*C_a_* (aF/μm^2^)	*σ_Ca_* (aF/μm^2^)	*σ_ε_* (aF/μm)	*t* (μm)	*u_c_*^3^ (μm)
1	p1	sub	*t_fox(p1/sub)elec_*	39.0	0.12	0.1	0.8846	0.0052
2	p1	aan	*t_thin(p1/aan)elec_*	1102.3	4.07	0.1	0.03130	0.00020
3	p2	sub	*t_fox(p2/sub)elec_*	38.8	0.10	0.1	08892	0.0050
4	p2	aan	*t_thin(p2/aan)elec_*	707.3	3.49	0.1	0.04878	0.00034
5	p2	p1	*t_thin(p2/p1)elec_*	579.6	1.35	0.1	0.05952	0.00033
6	m1	sub	[*t_fox,m1(pmd/sub)_* + *t_pmd(m1/fox)_*]*_elec_*	24.7	0.09	0.1	1.3968	0.0087
7	m1	aan	*t_pmd(m1/aan)elec_*	51.3	0.40	0.1	0.6725	0.0062
8	m1	p1	*t_pmd(m1/p1)elec_*	45.9	0.19	0.1	0.7516	0.0049
9	m1	p2	*t_pmd(m1/p2)elec_*	46.6	0.37	0.1	0.7403	0.0070
10	m2	sub	[*t_fox,m2(pmd/sub)_* + *t_pmd(imd/fox)_* + *_timd(m2/pmd)_*]*_elec_*	15.0	0.18	0.1	2.300	0.030
11	m2	aan	[*t_pmd(imd/aan)_* + *t_imd(m2/pmd)_*]*_elec_*	26.0	0.15	0.1	1.327	0.010
12	m2	p1	[*t_pmd(imd/p1)_*_+_ *t_imd(m2/pmd)_*]*_elec_*	22.6	0.24	0.1	1.527	0.018
13	m2	p2	[*t_pmd(imd/p2)_*_+_ *t_imd(m2/pmd)_*]*_elec_*	23.0	0.30	0.1	1.500	0.021
14	m2	m1	*t_imd(m2/m1)elec_*	37.4	0.36	0.1	0.922	0.010

1 Except for #2, #4, #7, and #11, the capacitors are over field oxide.

2 All capacitors are designed within a *p*-well region. The top electrode of the area capacitors is 240 μm by 300 μm.

3 See Sec. A.5 for calculation specifics.

**Table 7:** Thickness values for the interconnects

#	Symbol	*R_s_* (Ω/□)	*σ_Rs_* (Ω/□)	*ρ* (Ω μm)	*σ_ρ_* (Ω μm)	*t* (μm)	*u_c_*^1^ (μm)
1	*t_(p1)elec_*	27.4	0.38	8.5	0.1	0.3102	0.0076
2	*t_(p2)elec_*	19.6	0.22	7.0	0.1	0.3571	0.0097
3	*t_(m1)elec_*	0.0527	0.0023	0.033	0.001	0.626	0.043
4	*t_(m2)elec_*	0.0301	0.0014	0.032	0.001	1.063	0.076

1 See Sec. A.5 for calculation specifics.

**Table 8:** Summary of thickness values^1^

#	Thickness designation	*t_phys_* (µm)	*u_c,phys_* (µm)	Eq #	*t_elec_* (µm)	*u_c,elec_* (µm)	ref^2^ #
1	*t_fox(p1/sub)_*	0.8846^3^	0.0052	(16)	0.8846	0.0052	T6#1
2	*t_fox(p2/sub)_*	0.8697	0.0116	(32)	0.8892	0.0050	T6#3
3a	*t_fox,m1(pmd/sub)_*	0.8142	0.0115	(35)	0.7243	0.0107	(11)
3b	*t_fox,m2(pmd/sub)_*	0.8259	0.0113	(38)	0.973	0.035	(12)
4	*t_thin(p1/aan)_*	0.03130^3^	0.00020	(14)	0.03130	0.00020	T6#2
5	*t_thin(p2/aan)_*	0.04878^3^	0.00034	(15)	0.04878	0.00034	T6#4
6	*t_thin(p2/p1)_*	0.222	0.035	(50)	0.05952	0.00033	T6#5
7a	*t_pmd(m1/aan)_*	0.660	0.026	(47)	0.6725	0.0062	T6#7
7b		0.6797	0.0122	(48)	–	–	–
8a	*t_pmd(m1/fox)_*	0.660	0.026	(21)	0.6725	0.0062	(9)
8b		0.6797	0.0122	(21)	–	–	–
9	*t_pmd(m1/p1)_*	0.7516^3^	0.0049	(17)	0.7516	0.0049	T6#8
10	*t_pmd(m1/p2)_*	0.7561	0.0135	(49)	0.7403	0.0070	T6#9
11	*t_pmd(imd/aan)_*	0.405^3^	0.014	(43)	0.405	0.014	(5)
12	*t_pmd(imd/fox)_*	0.405^3^	0.014	(46)	0.405	0.014	(10)
13	*t_pmd(imd/p1)_*	0.465	0.017	(44)	0.605	0.021	(6)
14	*t_pmd(imd/p2)_*	0.470	0.018	(45)	0.578	0.023	(7)
15	*t_imd(m2/m1)_*	0.922	0.010	(19)	0.922	0.010	T6#14
16	*t_imd(m2/pmd)_*	0.922^3^	0.010	(20)	0.922	0.010	(4)
17	*t_imd(gl/m1)_*	0.835	0.081	(56)	–	–	–
18	*t_imd(gl/pmd)_*	0.835	0.081	(57)	–	–	–
19	*t_(p1)_*	0.3966	0.0080	(52)	0.3102	0.0076	T7#1
20	*t_(p1′)_*	0.3687	0.0079	(51)	0.2838	0.0077	(53)
21	*t_(p2)_*	0.4434	0.0159	(54)	0.3571	0.0097	T7#2
22	*t_(m1)_*	0.456	0.020	(55)	0.626	0.043	T7#3
23	*t_(m2)_*	1.085	0.085	(58)	1.063	0.076	T7#4
24	*t_(gl)_*	0.4876	0.0087	(63)	–	–	–
25	*t_(ni)_*	0.692	0.017	(64)	–	–	–

1 The highlighted entries correspond to the preferred values, as determined by the lower value of *u_c_*.

2 A reference number is recorded in this column. This could be a thickness equation number or a table entry. For example, (4) refers to equation (4) and T6#3 refers to entry #3 in Table 6.

3 The electrical value was the original source for these values.

**Table 9:** Equated oxide thicknesses

	Oxides being equated	*t* (μm)	*u_c_* (μm)	Ref^1^ #
1a	*t_imd(m2/pmd)elec_* = *t_imd(m2/m1)elec_*	0.922	0.010	T6#14 & (4)
1b	*t_imd(m2/m1)phys_* = *t_imd(m2/m1)elec_*	0.922	0010	(19) & T6#14
1c	*t_imd(m2/pmd)phys_* = *t_imd(m2/m1)phys_*	0.922	0.010	(20)
2	*t_imd(gl/pmd)phys_* = *t_imd(gl/m1)phys_*	0.835	0.081	(8) & (56)
3a	*t_pmd(m1/fox)elec_* = *t_pmd(m1/aan)elec_*	0.6725	0.0062	T6#7 & (9)
3b	*t_pmd(m1/fox)phys_* = *t_pmd(m1/aan)phys_*	0.660	0.026	(21) & (47)
3c		0.6797	0.0122	(48)
4a	*t_pmd(imd/fox)elec_* = *t_pmd(imd/aan)elec_*	0.405	0.014	(5) & (10)
4b	*t_pmd(imd/fox)phys_* = *t_pmd(imd/aan)phys_*	0.405	0.014	(22) & (43) & (46)
4c	*t_pmd(m1/p1)phys_* = *t_pmd(m1/p1)elec_*	0.7516	0.0049	(17) & T6#8
4d	[*t_pmd(imd/aan)_* + *t_imd(m2/pmd_)*]*_phys_* = [*t_pmd(imd/aan)_* + *t_imd(m2/pmd)_*]*_elec_*	1.327	0.010	(18) & T6#11 & (40)
5a	*t_fox,be(p1/sub)elec_* = *t_fox,be(p2/sub)elec_*	0.3919	0.0135	(30) & (13)
5b	= *t_fox,be,m1(pmd/sub)elec_*	0.3919	0.0135	(30) & (13)
5c	= *t_fox,be,m2(pmd/sub)elec_*	0.3919	0.0135	(30) & (13)
6a	*t_fox,be(p1/sub)phys_* = *t_fox,be(p2/sub)phys_*	0.4141	0.0103	(28) & (13)
6b	= *t_fox,be,m1(pmd/sub)phys_*	0.4141	0.0103	(28) & (13)
6c	= *t_fox,be,m2(pmd/sub)phys_*	0.4141	0.0103	(28) & (13)
7	*t_thin(p1/aan)phys_* = *t_thin(p1/aan)elec_*	0.03130	0.00020	T6#2 & (14)
8	*t_thin(p2/aan)phys_* = *t_thin(p2/aan)elec_*	0.04878	0.00034	T6#4 & (15)
9	*t_fox(p1/sub)phys_* = *t_fox(p1/sub)elec_*	0.8846	0.0052	T6#1 & (16)

1 A reference number or numbers are recorded in this column. These could be thickness equation numbers or table entry numbers. For example, (4) refers to equation (4) and T6#7 refers to entry #7 in Table 6.

**Table 10:** Comparing some field oxide thickness values^1^

	Thickness designation	*t_phys_* (μm)	*u_c,phys_* (μm)	Eq #	*t_elec_* (μm)	*u_c,elec_* (μm)	Eq #
1	*t_fox,be(p1/sub)_*	0.4141	0.0103	(28)	0.3919^2^	0.0135	(30)
2	*t_fox,ab(p1/sub)_*	0.4705	0.0089	(27)	0.4927^2^	0.0136	(29)
3	*t_fox,ab(p2/sub)_*	0.4556	0.0053	(31)	0.4973	0.0144	(33)
4	*t_fox,ab,m1(pmd/sub)_*	0.4001	0.0051	(34)	0.3324	0.0172	(36)
5	*t_fox,ab,m2(pmd/sub)_*	0.4118	0.0047	(37)	0.581	0.037	(39)

1 The highlighted entries correspond to the preferred values, as determined by the lower value of *u_c_*.

2 These values were obtained from crystal lattice calculations.

**Table 11:** Rank-ordering of *u_c_* values for the given thicknesses^1^

#	Thickness designation	*t_phys_* (µm)	*u_c,phys_* (µm)	*t_elec_* (µm)	*u_c,elec_* (µm)	*E_n_*
1	*t_thin(p1/aan)_*	0.03130	0.00020	0.03130	0.00020	–
2	*t_thin(p2/p1)_*	0.222	0.035	0.05952	0.00033	2.321
3	*t_thin(p2/aan)_*	0.04878	0.00034	0.04878	0.00034	–
4	*t_pmd(m1/p1)_*	0.7516	0.0049	0.7516	0.0049	–
5	*t_fox(p2/sub)_*	0.870	0.012	0.8892	0.0050	0.772
6	*t_fox(p1/sub)_*	0.8846	0.0052	0.8846	0.0052	–
7	*t_pmd(m1/aan)_*	0.680	0.012	0.6725	0.0062	0.263
8	*t_pmd(m1/fox)_*	0.680	0.012	0.6725	0.0062	0.263
9	*t_pmd(m1/p2)_*	0.756	0.014	0.7403	0.0070	0.520
10	*t_(p1)_*	0.3966	0.0080	0.3102	0.0076	3.915
11	*t_(p1′)_*	0.3687	0.0079	0.2838	0.0077	3.848
12	*t_fox,m1(pmd/sub)_* + *t_pmd(m1/fox)_*	1.494	0.017	1.3968	0.0087	2.566
13	*t_(gl)_*	0.4876	0.0087	–	–	–
14	*t_(p2)_*	0.443	0.016	0.3571	0.0097	2.317
15	*t_imd(m2/m1)_*	0.922	0.010	0.922	0.010	–
16	*t_imd(m2/pmd)_*	0.922	0.010	0.922	0.010	–
17	*t_pmd(imd/aan)_* + *t_imd(m2/pmd)_*	1.327	0.010	1.327	0.010	–
18	*t_fox,m1(pmd/sub)_*	0.814	0.012	0.724	0.011	2.862
19	*t_fox,m2(pmd/sub)_*	0.826	0.011	0.973	0.035	2.000
20	*t_pmd(imd/aan)_*	0.405	0.014	0.405	0.014	–
21	*t_pmd(imd/fox)_*	0.405	0.014	0.405	0.014	–
22	*t_pmd(imd/p1)_* + *t_imd(m2/pmd)_*	1.387	0.014	1.527	0.018	3.070
23	*t_pmd(imd/p2)_* + *t_imd(m2/pmd)_*	1.392	0.015	1.500	0.021	2.092
24	*t_fox,m2(pmd/sub)_* + *t_pmd(imd/fox)_* + *t_imd(m2/pmd)_*	2.153	0.015	2.300	0.030	2.191
25	*t_pmd(imd/p1)_*	0.465	0.017	0.605	0.021	2.591
26	*t_(ni)_*	0.692	0.017	–	–	–
27	*t_pmd(imd/p2)_*	0.470	0.018	0.578	0.023	1.849
28	*t_(m1)_*	0.456	0.020	0.626	0.043	1.796
29	*t_(m2)_*	1.085	0.085	1.063	0.076	0.096
30	*t_imd(gl/pmd)_*	0.835	0.081	–	–	–
31	*t_imd(gl/m1)_*	0.835	0.081	–	–	–

1 The rank-ordering is from the smallest to the largest *u_c_* value when looking at the highlighted entries. The highlighted entries correspond to the preferred values, as determined by the lower value of *u_c_*.

**Table 12:** Rank-ordering of *u_c_* values for the virtual oxide thicknesses^1^

#	Thickness designation	*t_phys_* (µm)	*u_c,phys_* (µm)	*t_elec_* (µm)	*u_c,elec_* (µm)	*E_n_*
1	*t_thin,be(p1/aan)_*	0.01465	0.00048	0.01387	0.00048	0.575
2	*t_thin,ab(p1/aan)_*	0.01665	0.00048	0.01743	0.00048	0.575
3	*t_thin,be(p2/aan)_*	0.02283	0.00075	0.02161	0.00075	0.575
4	*t_thin,ab(p2/aan)_*	0.02595	0.00075	0.02717	0.00076	0.571
5	*t_thin,be(p2/p1)_*	0.104	0.017	0.02637	0.00090	2.280
6	*t_thin,ab(p2/p1)_*	0.118	0.019	0.03315	0.00091	2.230
7	*t_fox,ab,m2(pmd/sub)_*	0.4118	0.0047	0.581	0.037	2.270
8	*t_fox,ab,m1(pmd/sub)_*	0.4001	0.0051	0.332	0.017	1.887
9	*t_fox,ab(p2/sub)_*	0.4556	0.0053	0.497	0.014	1.359
10	*t_fox,ab(p1/sub)_*	0.4705	0.0089	0.493	0.014	0.683
11	*t_fox,be(p1/sub)_*	0.414	0.010	0.392	0.014	0.654

1 The rank-ordering is from the smallest to the largest *u_c_* value when looking at the highlighted entries. The highlighted entries correspond to the preferred values, as determined by the lower value of *u_c_*.

**Table 13:** Rank-ordering of thickness values^1^

#	Thickness designation	*t_phys_* (µm)	*u_c,phys_* (µm)	*t_elec_* (µm)	*u_c,elec_* (µm)	*E_n_*
1	*t_thin(p1/aan)_*	0.03130	0.00020	0.03130	0.00020	–
2	*t_thin(p2/aan)_*	0.04878	0.00034	0.04878	0.00034	–
3	*t_thin(p2/p1)_*	0.222	0.035	0.05952	0.00033	2.321
4	*t_(p1′)_*	0.3687	0.0079	0.2838	0.0077	3.848
5	*t_(p1)_*	0.3966	0.0080	0.3102	0.0076	3.915
6	*t_(p2)_*	0.443	0.016	0.3571	0.0097	2.317
7	*t_pmd(imd/fox)_*	0.405	0.014	0.405	0.014	–
8	*t_pmd(imd/aan)_*	0.405	0.014	0.405	0.014	–
9	*t_(m1)_*	0.456	0.020	0.626	0.043	1.796
10	*t_pmd(imd/p1)_*	0.465	0.017	0.605	0.021	2.591
11	*t_pmd(imd/p2)_*	0.470	0.018	0.578	0.023	1.849
12	*t_(gl)_*	0.4876	0.0087	–	–	–
13	*t_pmd(m1/fox)_*	0.680	0.012	0.6725	0.0062	0.263
14	*t_pmd(m1/aan)_*	0.680	0.012	0.6725	0.0062	0.263
15	*t_(ni)_*	0.692	0.017	–	–	–
16	*t_fox,m1(pmd/sub)_*	0.814	0.012	0.724	0.011	2.862
17	*t_pmd(m1/p2)_*	0.756	0.014	0.7403	0.0070	0.520
18	*t_pmd(m1/p1)_*	0.7516	0.0049	0.7516	0.0049	–
19	*t_fox,m2(pmd/sub)_*	0.826	0.011	0.973	0.035	2.000
20	*t_imd(gl/pmd)_*	0.835	0.081	–	–	–
21	*t_imd(gl/m1)_*	0.835	0.081	–	–	–
22	*t_fox(p1/sub)_*	0.8846	0.0052	0.8846	0.0052	–
23	*t_fox(p2/sub)_*	0.870	0.012	0.8892	0.0050	0.772
24	*t_imd(m2/pmd)_*	0.922	0.010	0.922	0.010	–
25	*t_imd(m2/m1)_*	0.922	0.010	0.922	0.010	–
26	*t_(m2)_*	1.085	0.085	1.063	0.076	0.096
27	*t_pmd(imd/aan)_* + *t_imd(m2/pmd)_*	1.327	0.010	1.327	0.010	–
28	*t_pmd(imd/p1)_* + *t_imd(m2/pmd)_*	1.387	0.014	1.527	0.018	3.070
29	*t_pmd(imd/p2)_* + *t_imd(m2/pmd)_*	1.392	0.015	1.500	0.021	2.092
30	*t_fox,m1(pmd/sub)_* + *t_pmd(m1/fox)_*	1.494	0.017	1.3968	0.0087	2.566
31	*t_fox,m2(pmd/sub)_* + *t_pmd(imd/fox)_* + *t_imd(m2/pmd)_*	2.153	0.015	2.300	0.030	2.191

1 The rank-ordering is from the smallest to the largest thickness value when looking at the highlighted entries. The highlighted entries correspond to the preferred values, as determined by the lower value of *u_c_*.

**Table 14:** Rank-ordering of capacitance values from Table 6 1

#	Thickness designation	*C_a_* (aF/μm^2^)	*σ_Ca_*(aF/μm^2^)	*σ_ε_* (aF/μm)	*t_elec_* (μm)	*u_c,elec_* (μm)	*t_phys_* (μm)	*u_c,phys_* (μm)
1	*t_thin(p1/aan)_*	1102.3	4.07	0.1	0.03130	0.00020	0.03130	0.00020
2	*t_thin(p2/aan)_*	707.3	3.49	0.1	0.04878	0.00034	0.04878	0.00034
3	*t_thin(p2/p1)_*	579.6	1.35	0.1	0.05952	0.00033	0.222	0.035
4	*t_pmd(m1/aan)_*	51.3	0.40	0.1	0.6725	0.0062	0.680	0.012
5	*t_pmd(m1/p2)_*	46.6	0.37	0.1	0.7403	0.0070	0.756	0.014
6	*t_pmd(m1/p1)_*	45.9	0.19	0.1	0.7516	0.0049	0.7516	0.0049
7	*t_fox(p1/sub)_*	39.0	0.12	0.1	0.8846	0.0052	0.8846	0.0052
8	*t_fox(p2/sub)_*	38.8	0.10	0.1	0.8892	0.0050	0.870	0.012
9	*t_imd(m2/m1)_*	37.4	0.36	0.1	0.922	0.010	0.922	0.010
10	*t_pmd(imd/aan)_* + *t_imd(m2/pmd)_*	26.0	0.15	0.1	1.327	0.010	1.327	0.010
11	*t_fox,m1(pmd/sub)_* + *t_pmd(m1/fox)_*	24.7	0.09	0.1	1.3968	0.0087	1.494	0.017
12	*t_pmd(imd/p2)_* + *t_imd(m2/pmd)_*	23.0	0.30	0.1	1.500	0.021	1.392	0.015
13	*t_pmd(imd/p1)_* + *t_imd(m2/pmd)_*	22.6	0.24	0.1	1.527	0.018	1.387	0.014
14	*t_fox,m2(pmd/sub)_* + *t_pmd(imd/fox)_* + *t_imd(m2/pmd)_*	15.0	0.18	0.1	2.300	0.030	2.153	0.015

1 The highlighted entries correspond to the preferred values, as determined by the lower value of *u_c_*.

**Table 15:** Step height measurements from Table 5 with an additional uncertainty component

#	step*	Height (μm)	*σ_platNX_* (μm)	*σ_platNY_* (μm)	*u_basic_* (μm)	*u_Wstep_* (μm)	*u_Lstep_* (μm)	*u_c_* (μm)
1	*step1_AB_*	0.454	0.0077	0.0029	0.0046	0.0076	0.0052	0.010
2	*step1_CD_*	−0.4296	0.0048	0.0025	0.0044	0.0027	0.0023	0.0056
3	*step1_EF_*	0.4001	0.0040	0.0040	0.0041	0.0031	0.0021	0.0056
4	*step1_GH_*	−0.4118	0.0070	0.0052	0.0042	0.0021	0.0052	0.0070
5	*step1_rA_*	0.387	0.0048	0.0077	0.0040	0.0032	0.0057	0.008
6	*step1_rD_*	0.4477	0.0048	0.0025	0.0046	0.0027	0.0023	0.0058
7	*step1_rE_*	0.7113	0.0048	0.0040	0.0073	0.0050	0.0027	0.0092
8	*step2_rA_*	0.730	0.0048	0.0084	0.0075	0.0036	0.0063	0.010
9	*step2_AB_*	0.399	0.0084	0.0049	0.0041	0.0071	0.0064	0.010
10	*step2_BC_*	0.480	0.0049	0.0033	0.0049	0.0045	0.0025	0.007
11	*step2_CD_*	−0.419	0.0033	0.0081	0.0043	0.0065	0.0057	0.010
12	*step2_BD_*	0.0607	0.0049	0.0081	0.0006	0.0021	0.0061	0.0065
13	*step3_AB_(0)*	1.180	0.0106	0.0048	0.0121	0.0081	0.0084	0.017
14	*step3_AB_(4)*	0.488	0.0084	0.0047	0.0050	0.0071	0.0063	0.011
15	*step3_BC_(0)*	−0.419	0.0048	0.0049	0.0043	0.0137	0.0033	0.015

**Table 16:** Rank-ordering of capacitance values from Table 14 with an additional uncertainty component^1^

#	Thickness designation	*C_a_* (aF/µm^2^)	*t_elec_* (µm)	*u_c,elec,old_* (µm)	*σ_resCa_* (aF/µm^2^)	*u_res_*^2^ (µm)	*u_c,elec_* (µm)	*t_phys_* (µm)	*u_c,phys_* (µm)
1	*t_thin(p1/aan)_*	1102.3	0.03130	0.00020	1.5	0.000043	0.00020	0.03130	0.00020
2	*t_thin(p2/aan)_*	707.3	0.04878	0.00034	1.5	0.000103	0.00036	0.04878	0.00036
3	*t_thin(p2/p1)_*	579.6	0.05952	0.00033	1.5	0.000154	0.00036	0.222	0.035
4	*t_pmd(m1/aan)_*	51.3	0.6725	0.0062	1.5	0.019664	0.0206	0.680	0.012
5	*t_pmd(m1/p2)_*	46.6	0.7403	0.0070	1.5	0.023829	0.0248	0.756	0.014
6	*t_pmd(m1/p1)_*	45.9	0.7516	0.0049	1.5	0.024562	0.0250	0.7516	0.0250
7	*t_fox(p1/sub)_*	39.0	0.8846	0.0052	1.5	0.034023	0.0344	0.8846	0.0344
8	*t_fox(p2/sub)_*	38.8	0.8892	0.0050	1.5	0.034376	0.0347	0.870	0.012
9	*t_imd(m2/m1)_*	37.4	0.922	0.010	1.5	0.036979	0.038	0.922	0.038
10	*t_pmd(imd/aan)_* + *t_imd(m2/pmd)_*	26.0	1.327	0.010	1.5	0.076558	0.077	1.327	0.077
11	*t_fox,m1(pmd/sub)_* + *t_pmd(m1/fox)_*	24.7	1.397	0.016	1.5	0.084826	0.086	1.494	0.017
12	*t_pmd(imd/p2)_* + *t_imd(m2/pmd)_*	23.0	1.500	0.021	1.5	0.097826	0.100	1.392	0.015
13	*t_pmd(imd/p1)_* + *t_imd(m2/pmd)_*	22.6	1.527	0.022	1.5	0.101350	0.104	1.387	0.014
14	*t_fox,m2(pmd/sub)_* + *t_pmd(imd/fox)_* + *t_imd(m2/pmd)_*	15.0	2.300	0.044	1.5	0.230000	0.234	2.153	0.015

1 The highlighted entries correspond to the preferred values, as determined by the lower value of *u_c_*.

2 Where *u_res_* = *σ_resCa_ t_elec_*_/_*Ca*

**Table 17:** Revised rank-ordering of *u_c_* values for the given thicknesses^1^

#	Thickness designation	*t_phys_* (µm)	*u_c,phys_* (µm)	*t_elec_* (µm)	*u_c,elec_* (µm)	*E_n_*
1	*t_thin(p1/aan)_*	0.03130	0.00020	0.03130	0.00020	–
2	*t_thin(p2/p1)_*	0.222	0.075	0.05952	0.00036	1.083
3	*t_thin(p2/aan)_*	0.04878	0.00036	0.04878	0.00036	–
4	*t_(p1)_*	0.3966	0.0097	0.3102	0.0076	3.506
5	*t_(p1′)_*	0.3687	0.0097	0.2838	0.0077	3.428
6	*t_(p2)_*	0.443	0.039	0.3571	0.0097	1.082
7	*t_(gl)_*	0.488	0.011	–	–	–
8	*t_(ni)_*	0.692	0.020	–	–	–
9	*t_pmd(m1/aan)_*	0.680	0.028	0.673	0.021	0.103
10	*t_pmd(m1/fox)_*	0.680	0.028	0.673	0.021	0.103
11	*t_pmd(m1/p1)_*	0.752	0.025	0.752	0.025	–
12	*t_pmd(m1/p2)_*	0.756	0.029	0.740	0.025	0.208
13	*t_fox(p1/sub)_*	0.885	0.034	0.885	0.034	–
14	*t_fox(p2/sub)_*	0.870	0.036	0.889	0.035	0.194
15^2^	*t_fox,m1(pmd/sub)_*	0.814	0.036	0.724	0.088	0.473
16^2^	*t_fox,m2(pmd/sub)_*	0.826	0.037	0.97	0.25	0.291
17	*t_imd(m2/m1)_*	0.922	0.038	0.922	0.038	–
18	*t_imd(m2/pmd)_*	0.922	0.038	0.922	0.038	–
19	*t_(m1)_*	0.456	0.091	0.626	0.043	0.846
20	*t_fox,m1(pmd/sub)_* + *t_pmd(m1/fox)_*	1.494	0.046	1.397	0.085	0.501
21	*t_(m2)_*	1.09	0.15	1.063	0.076	0.065
22	*t_pmd(imd/aan)_* + *t_imd(m2/pmd)_*	1.327	0.077	1.327	0.077	–
23	*t_pmd(imd/p1)_* + *_timd(m2/pmd)_*	1.387	0.078	1.53	0.10	0.542
24	*t_pmd(imd/p2)_* + *t_imd(m2/pmd)_*	1.392	0.078	1.50	0.10	0.426
25	*t_fox,m2(pmd/sub)_* + *t_pmd(imd/fox)_* + *t_imd(m2/pmd)_*	2.153	0.085	2.30	0.23	0.297
26	*t_pmd(imd/aan)_*	0.405	0.086	0.405	0.086	–
27	*t_pmd(imd/fox)_*	0.405	0.086	0.405	0.086	–
28	*t_pmd(imd/p2)_*	0.470	0.087	0.58	0.11	0.392
29	*t_pmd(imd/p1)_*	0.465	0.087	0.61	0.11	0.499
30	*t_imd(gl/pmd)_*	0.84	0.15	–	–	–
31	*t_imd(gl/m1)_*	0.84	0.15	–	–	–

1 The rank-ordering is from the smallest to the largest *u_c_* value when looking at the highlighted entries. The highlighted entries correspond to the preferred and reported values, as determined by the lower value of *u_c_*.

2 This table indicates that if *t_fox(pmd/sub)_* = *t_fox,m1(pmd/sub)_* = *t_fox,m2(pmd/sub)_*, then *t_phys_* for #15 is preferred.

**Table 18:** Revised rank-ordering of *u_c_* values for the virtual oxide thicknesses^1^

#	Thickness designation	*t_phys_* (µm)	*u_c,phys_* (µm)	*t_elec_* (µm)	*u_c,elec_* (µm)	*E_n_*
1	*t_thin,be(p1/aan)_*	0.01465	0.00048	0.01387	0.00048	0.575
2	*t_thin,ab(p1/aan)_*	0.01665	0.00048	0.01743	0.00048	0.575
3	*t_thin,be(p2/aan)_*	0.02283	0.00075	0.02161	0.00075	0.575
4	*t_thin,ab(p2/aan)_*	0.02595	0.00076	0.02717	0.00076	0.568
5	*t_thin,be(p2/p1)_*	0.104	0.035	0.02637	0.00091	1.109
6	*t_thin,ab(p2/p1)_*	0.118	0.040	0.03315	0.00092	1.060
7	*t_fox,ab,m1(pmd/sub)_*	0.4001	0.0056	0.332	0.090	0.375
8	*t_fox,ab(p2/sub)_*	0.4556	0.0057	0.497	0.040	0.514
9	*t_fox,ab,m2(pmd/sub)_*	0.4118	0.0070	0.58	0.25	0.337
10	*t_fox,ab(p1/sub)_*	0.471	0.010	0.493	0.023	0.436
11	*t_fox,be(p1/sub)_*	0.414	0.036	0.392	0.020	0.269

1 The rank-ordering is from the smallest to the largest *u_c_* value when looking at the highlighted entries. The highlighted entries correspond to the preferred and reported values, as determined by the lower value of *u_c_*.

## Footnotes

2 Certain commercial equipment, instruments, or materials are identified in this paper to foster understanding. Such identification does not imply recommendation or endorsement, nor does it imply that the equipment identified are necessarily the best available for the purpose.

3 These 25 thicknesses correspond to the thicknesses in the 48 layer stacks mentioned in the introduction.

4 In Table 2, the additional nomenclature associated with the platforms in TS #3 is explained in a note beneath the table.

5 If one 3-D data set is used to obtain the step-height measurement, three 2-D data traces along the step are extracted from the 3-D data set. The platform measurements obtained from each trace are subtracted from each other after which the results are averaged to find *step1_CD_*, as specified in the appendix [12]. For this case, the resulting value for *step1_CD_* is given in Table 5.

6 A dielectric is an electrically nonconducting material, such as an oxide.

7 Although crystal lattice calculations can result in what can be considered a “physical” quantity, we are viewing these calculations as part of the electrical approach to more sharply distinguish between the interferometric measurements and calculations used in the physical approach and the non-interferometric measurements and calculations used in the electrical approach.

8 The designation for the reduced, oxidized thickness of p1 after the creation of *t_thin(p2/p1)_* is given by *t_(p1_*_′_*_)_*.

9 In actuality, numbers 41 to 48 can be considered; however, the numbers mentioned have the greatest potential of producing the lowest value for *u_c_* for *h_AA_*.

10 The *u_c_* value for the physical approach in #7a of Tables 8 is used in these calculations.

11 It could be worth exploring the possibility of a step similar to *step2_CD_* but without the m1 present.

12 The appendix points out that it is preferable to use *step3_CD_(0)* instead of *plat3D(0)* minus *plat3C(0)*. However, since two different data sets in two different data sessions were used to find *plat3D(0)* and *plat3C(0)*, this approach is being taken.

13 A 20× magnification was used for the data set, having a field of view of about 600 μm by 450 μm.

14 A data session typically does not last longer than 3 hours. Therefore, if interferometric data is taken on two different days, then this corresponds to two different data sessions.

15 For the processing run from which the test chips were obtained, *σ_Ca_* is the standard deviation of 18 measurements with 9 measurements taken on 9 different test chips across one wafer and 9 measurements taken on 9 different test chips across the second wafer. There were only two wafers in this processing run.
